# Assessment of Amide proton transfer weighted (APTw) MRI for pre-surgical prediction of final diagnosis in gliomas

**DOI:** 10.1371/journal.pone.0244003

**Published:** 2020-12-29

**Authors:** Faris Durmo, Anna Rydhög, Frederik Testud, Jimmy Lätt, Benjamin Schmitt, Anna Rydelius, Elisabet Englund, Johan Bengzon, Peter van Zijl, Linda Knutsson, Pia C. Sundgren

**Affiliations:** 1 Division of Radiology, Department of Clinical Sciences, Lund University, Lund, Sweden; 2 Center for Medical Imaging and Physiology, Skåne University Hospital, Lund, Sweden; 3 Siemens Healthcare AB, Malmö, Sweden; 4 Siemens Healthcare Pty Ltd, Bayswater, Australia; 5 Division of Neurology, Department of Clinical Sciences, Lund University, Lund, Sweden; 6 Division of Oncology and Pathology, Department of Clinical Sciences, Lund University, Lund, Sweden; 7 Division of Neurosurgery, Department of Clinical Sciences, Lund University, Lund, Sweden; 8 Russell H. Morgan Department of Radiology and Radiological Science, Johns Hopkins University School of Medicine, Baltimore, MD, United States of America; 9 F.M. Kirby Research Center for Functional Brain Imaging, Kennedy Krieger Institute, Baltimore, MD, United States of America; 10 Department of Medical Radiation Physics, Lund University, Lund, Sweden; 11 LBIC, Lund University Bioimaging Center, Lund University, Lund, Sweden; Vanderbilt University, UNITED STATES

## Abstract

**Purpose:**

Radiological assessment of primary brain neoplasms, both high (HGG) and low grade tumors (LGG), based on contrast-enhancement alone can be inaccurate. We evaluated the radiological value of amide proton transfer weighted (APTw) MRI as an imaging complement for pre-surgical radiological diagnosis of brain tumors.

**Methods:**

Twenty-six patients were evaluated prospectively; (22 males, 4 females, mean age 55 years, range 26–76 years) underwent MRI at 3T using T1-MPRAGE pre- and post-contrast administration, conventional T2w, FLAIR, and APTw imaging pre-surgically for suspected primary/secondary brain tumor. Assessment of the additional value of APTw imaging compared to conventional MRI for correct pre-surgical brain tumor diagnosis. The initial radiological pre-operative diagnosis was based on the conventional contrast-enhanced MR images. The range, minimum, maximum, and mean APTw signals were evaluated.

Conventional normality testing was performed; with boxplots/outliers/skewness/kurtosis and a Shapiro–Wilk’s test. Mann-Whitney U for analysis of significance for mean/max/min and range APTw signal. A logistic regression model was constructed for mean, max, range and Receiver Operating Characteristic (ROC) curves calculated for individual and combined APTw signals

**Results:**

Conventional radiological diagnosis prior to surgery/biopsy was HGG (8 patients), LGG (12 patients), and metastasis (6 patients). Using the mean and maximum: APTw signal would have changed the pre-operative evaluation the diagnosis in 8 of 22 patients (two LGGs excluded, two METs excluded). Using a cut off value of >2.0% for mean APTw signal integral, 4 of the 12 radiologically suspected LGG would have been diagnosed as high grade glioma, which was confirmed by histopathological diagnosis. APTw mean of >2.0% and max >2.48% outperformed four separate clinical radiological assessments of tumor type, P-values = .004 and = .002, respectively.

**Conclusions:**

Using APTw-images as part of the daily clinical pre-operative radiological evaluation may improve diagnostic precision in differentiating LGGs from HGGs, with potential improvement of patient management and treatment.

## Introduction

Glioblastoma has been and still is associated with a poor outcome for afflicted patients [1–3] with an overall survival rate below 6% [4]. It is also one of the most frequently occurring brain lesions [2,3] and can be, in the early stages of the disease, difficult to discern radiologically from low grade glioma [5], which have a better prognosis [6].

Even when using the current optimal treatment for glioblastoma, the STUPP protocol [1] consisting of gross total resection (GTR) with follow-up combined radiotherapy and chemotherapy (Temozolomide), the increase in survival is modest with regards to previous treatment regimens [7]. Moreover, there has not been a breakthrough in the reduction of recurrences after a few months of treatment [4]. The WHO classification update for the diagnosis of CNS tumors in 2016 has brought more emphasis on genetics and epigenetics. The new classification now categorizes glioblastomas into subgroups based on the immunoreactivity of isocitrate dehydrogenase (IDH): IDH-mutant with more favorable prognosis, IDH-wildtype with less favorable prognosis, and IDH-NOS (IDH-none other specified) [8]. Presently, it is known that glioblastomas can be of different genetic subtypes, arising from either progenitor cells, stem cells, or from de-differentiation of gliomas of grades 2 and 3 [9]. Most gliomas of WHO grade 1 and 2 which are resected tend to recur and it is evident that the recurrent tumor comes with a greater risk of malignant transformation into grades 3 and 4 [6]. Additionally, it has been suggested that malignant transformation in low grade glioma (LGG) ensues as a consequence of its natural course over time [10].

In the clinical setting, there are still issues with regards to the radiological assessment of primary brain neoplasms because conventional contrast-enhanced (gadolinium based) MRI for differentiation of LGG and high grade glioma (HGG) has only shown sensitivity and specificity ranging from 72.5–97% and 65–73%, respectively [11–14]. Furthermore, it is occasionally difficult to differentiate between brain tumors radiologically due to lack of enhancement, which is reflected in the fact that misclassifications can reach a false negative rate of 50% in patients with de novo debut of enhancing suspected brain neoplasms i.e. glioma [5]. Additionally, non-enhancing supratentorial lesions can be either LGG or HGG because up to 60% of lesions have been identified histologically as LGG and 40% as anaplastic astrocytoma i.e. HGG [15]. There is an apparent need for evaluating and validating novel techniques for pre-operatively differentiating not only non-enhancing but also enhancing malignant brain tumors such as HGG, LGG and metastases (MET).

One of the new MR techniques with demonstrated potential for differentiating non-enhancing and enhancing brain tumors is Amide proton transfer weighted (APTw) MRI [16,17]. APTw imaging [16,17] is a chemical exchange saturation transfer (CEST) technique [18] that utilizes exchange of the amide protons located in the peptide bonds of mobile proteins and peptides with the water used for MRI, which can be used as an indirect indicator of the tissue mobile protein content. Brain neoplasms have been shown to be suitable targets for identification with APTw MRI due to increased mobile protein content in comparison with normal appearing white matter [19] as well as a slightly increased intracellular pH [20]. APTw MRI can be easily performed on standard 3 T systems in the span of five minutes [21–23]. Additionally, the technique can identify tissue diversity and for instance help discern between necrosis, cystic compartments and viable tumor [24]. The technique has shown to help discriminate not just between well-defined cohorts of LGG and HGG [25] but also lymphomas from HGG [26]. Likewise, APTw signal has shown to increase with increasing glioma grade [27], predicts histopathological grading of diffuse gliomas [28] and is useful to differentiate diffuse gliomas manifesting without intense contrast enhancement [29]. If APTw imaging shall have a roll in the radiological routine and pre-surgical work-up of brain lesions we need to address the question—how well can APTw distinguish low and high grade gliomas in clinical routine where the final diagnosis in not known. Histologic tumor features are relevant for an integrated diagnosis and prognosis. Also, APT signal intensity corresponds to prognosis and overall survival [30].

The primary aim of this study was to evaluate if noninvasive APTw MRI can, in daily clinical routine, increase the radiological accuracy in differentiating less malignant tumors from more malignant ones, i.e. LGG from HGG prior to surgery. The second objective was to examine if discrepancies between radiological diagnosis and histopathological diagnosis occur to the same degree as previously reported in the literature.

## Materials and methods

### MRI acquisition protocol for APTw and conventional MRI sequences

The project was approved by the local ethics committee (The Regional Ethical Review Board in Lund (#2016/531, #2017/866, #2018/993), and written informed consent was obtained from each volunteer. The study initially consisted of 26 pre-surgical patients with brain lesions (mean age 55 years, range 26–76 years, 22 males, 4 females). Inclusion criteria were age >18, suspicion of brain neoplasm i.e. glioma WHO grades 1 to 4 or a suspected brain metastasis, (note that in this material, one case of WHO grade 3 being the exception, we only had WHO grade 2 or 4 gliomas). Patients were examined on a 3T scanner (MAGNETOM Prisma, Siemens Healthcare, Erlangen, Germany). APTw images were obtained using a CEST prototype sequence of the manufacturer with 3D GRE (22 slices, 2x2x4 mm^3^) acquisition of the water saturation spectrum (Z-spectrum), using B1 = 2 μT, 21 frequency offsets from -610 to 610 Hz plus an unsaturated reference image, referred to as S_0_.The S_0_ image was acquired with off-resonant pre-saturation frequency of -150 ppm relative to water to suppress the macromolecular background. The saturation module consisted of 5 hyperbolic secant pulses of 100 ms and four 61 ms interpulse delays. Total acquisition time for APTw MRI was 6:50 min. Conventional MRI sequences such as axial T2 2D Turbo spin-echo (TSE), axial 2D/3D fluid attenuated inversion recovery (FLAIR), and T1 3D magnetization prepared rapid gradient echo (MPRAGE) pre- and post-Gd contrast administration were obtained with the following isotropic resolutions: 1 mm (T1w MPRAGE and 3D FLAIR), 5 mm (T2 turbo spin-echo), and 3 mm (2D FLAIR). The sequence for APTw imaging was added as part of the research protocol to the conventional Gd-based MRI sequences and acquired before contrast administration. The complete examination time for the entire protocol was 45 minutes.

### Postprocessing of the APTw MRI

In order to calculate the APTw signal in percent one must first normalize the water signal during saturation at a frequency offset from water with the signal without saturation (S_0_). By doing this at multiple frequencies a so-called Z-spectrum is obtained [16,31]. After B0 correction (done automatically on the scanner using the bottom of the Z-spectrum), the APTw signal integral over the applicable amide proton range of the Z-spectrum was also calculated automatically in each voxel on the scanner as part of the prototype sequence. This integral calculation was referenced (by subtraction) to the integral at the opposite frequency relative to the water frequency (asymmetry analysis):

$$APTw={MTR}_{asym}^{Integr}(3.5\pm 0.4ppm)=\frac{\left\{P(-\Delta \omega )-P(+\Delta \omega )\right\}}{Deltafreq∙S_{0}}=[C(x,y)-2048]/(scalefactor∙2000)$$

in which P is a summation over the range Deltafreq (integral range) of 0.8 ppm in the Z-spectrum, and Δ*ω* the frequency offset at the center of the integral range. This is calculated from the signal C(x, y) of the voxel with scalefactor = 10.

For all subjects, pre-and postcontrast enhanced T1-MPRAGE, FLAIR, and APTw images were acquired within the same imaging protocol. Conventional MR sequences included: T1-MPRAGE; TR/TE 1900/2.54 milliseconds, FOV 256×256 mm^2^, acq. matrix 256×256, inversion time 900 milliseconds, TA 5:13. Also, FLAIR (3D) TR/TE 5000/393 milliseconds, FOV 256×256 mm^2^, acq. matrix 256×256, inversion time 1800 milliseconds, TA 4:25.

**Fig 1:**
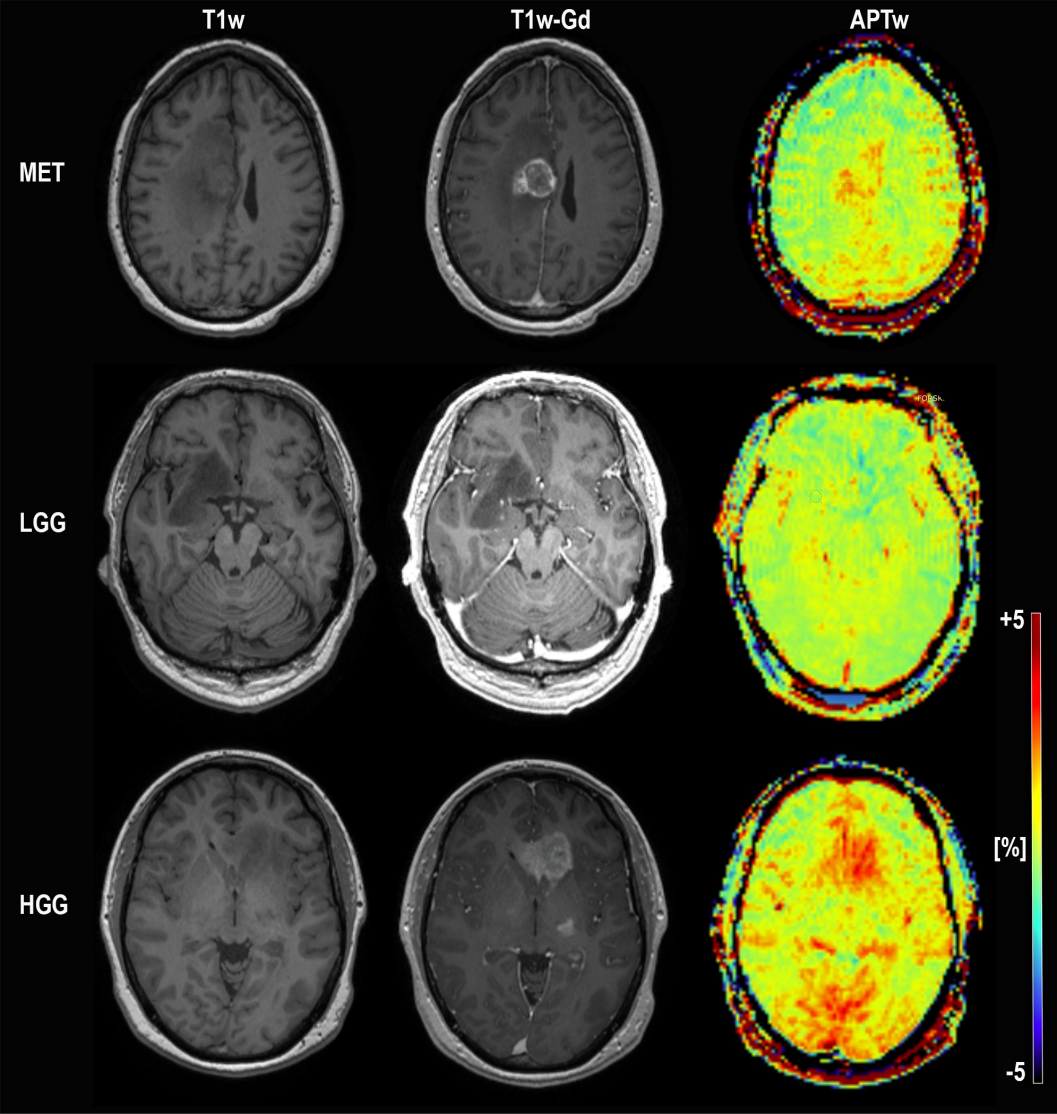
**Conventional MRI; T1-MPRAGE without contrast, T1-MPRAGE with Gadolinium and APTw in color for subject 25 (MET; top) and subject 17 (LGG; middle) and subject 18 (HGG; bottom).** The ROI placement for each subject is depicted. The APTw signal intensity for the high grade glioma and metastasis subject is distinctly higher than for the low grade glioma subject in the middle. Subject 25 MET (misdiagnosed by reader 2 and reader 3 as HGG, correctly diagnosed by APTw), Subject 17 LGG (correctly diagnosed by all). Subject 18 HGG (misdiagnosed by the initial radiological assessment as LGG, correctly diagnosed by APTw and all readers)”.

**Fig 2:**
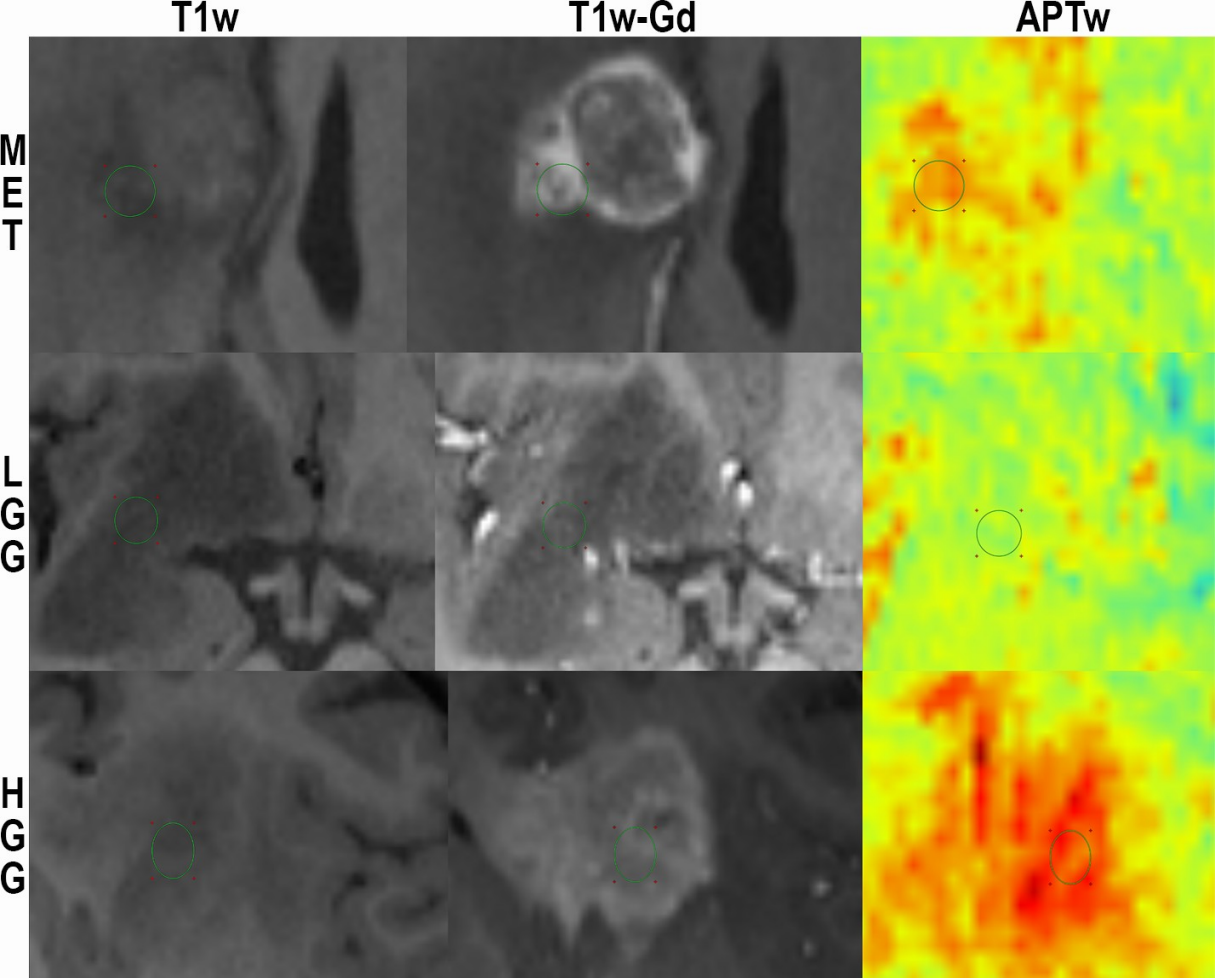
Enlargement of Fig 1 for visualization of ROI-placement. T1-MPRAGE without contrast, T1-MPRAGE with Gadolinium and APTw in color for subject 25 (MET; top) and subject 17 (LGG; middle) and subject 18 (HGG; bottom).

Images were extracted by an MR physicist blinded to the histopathological diagnosis for: 1) quantification of APTw signal by an M.D. and 2) concurrent and independent diagnostic review and analysis of the conventional images by two senior consultants in neuroradiology with over 20 years of experience (readers 1 and 2) and one junior consultant with 2 years of experience (reader 3). Each slice of the tumor was carefully assessed and each slice of pathological tissue correlated to T1-MPRAGE post contrast was probed in search for the highest APTw signal. We chose one ROI, placed in the area of highest signal intensity as show in (Figs 1 and 2). Recent APT papers have shown that the area of maximum APTw signal is representative of the tumor type and we followed this principle [32,33].

Gadolinium-enhanced, T1-MPRAGE and FLAIR images were then analyzed concomitantly with APTw images. Areas with signal abnormalities and highest APTw signal were chosen for placement of one circular region of interest (ROI) on APTw color maps, with a size of 10 pixels. The ROIs were placed in the contrast enhanced part of the lesion or, in cases without enhancement, in areas with tissue hypointensities on T1-MPRAGE overlapping anatomically with hyperintensities on FLAIR as previously described [24].

Mean, maximum, minimum and range (within subject difference between max APTw–min APTw) of the APTw signal were extracted. Postprocessing was performed in RadiAnt DICOM Viewer (Medixant, Poznan, Poland). A circular ROI was drawn manually and positioned on morphological T1-MPRGAE pre and post Gd-contrast images, with assistance of FLAIR images, using: Medixant. RadiAnt DICOM Viewer [Software], version 2020.1. Mar 9, 2020. URL: https://www.radiantviewer.com. For the S1 File. a whole lesion ROI was drawn manually and positioned on 1 slice of morphological T1-MPRGAE post Gd-contrast images, with assistance of FLAIR images, 3DSlicer 4.11.0, Nov 1, 2020. URL: http://www.slicer.org/ [34]. The ROIs were thereafter copied within the programs to the APTw maps and manually adjusted, if necessary, to achieve the same anatomical location as the selected ROI on the morphological image. This was to ensure that the ROIs were placed in the appropriate area of signal abnormality on each map. Special care was taken as not to place the ROI within area with hemorrhagic, cystic or necrotic content. Placements of the ROI were ascertained and validated by a senior neuroradiologist with over 20 years of experience (Dr P.C.S).

### Radiological evaluation

The radiological evaluation of the brain tumors diagnosis was performed in 3 steps: the initial radiological report; review of the conventional MR images by three external neuroradiologists (second review); and by finally adding the APTw images to the conventional MR images for assessment of the diagnosis.

More specifically, the initial clinical radiological evaluation and primary diagnosis were based on the conventional MR images. The initial radiological assessment was performed by senior radiologists, in case of junior radiologist or radiologist in training; all cases were co-assessed and co-signed by senior neuroradiologist. In addition to this, an assessment of the same images was performed by three external neuroradiologist (two with over 20 years’ experience and one with 2 years’ experience) (second review). They were given the same clinical information but were blinded to the final histopathological diagnosis. They evaluated the images and reported the final diagnosis either as LGG, HGG or MET, three common differential diagnoses in daily clinical routine. APTw imaging presently is not part of the routine pre-operative MR protocol for brain tumors and considered only as an add-on sequence for research purposes. It was therefore included only in the secondary phase of evaluation of the examinations. As the APTw images were part of a research protocol, they were initially not analyzed by either the clinical general radiologist or neuroradiologist at time of the initial report of the study or by the three external neuroradiologists reviewing the study. The APTw signals were evaluated in the third phase to verify if the pre-surgical diagnosis would have changed if the APTw measurements had been included in the initial radiological assessment. However, the diagnosis in some cases is easier to define based on underlying clinical information i.e. known non-CNS cancer etc. For example, in our study the initial radiological diagnosis for six patients with single lesions was metastasis (MET), while only two patients were histopathologically proven to be MET. Therefore, based on limited material of patients with proven MET, it was decided to not evaluate HGG versus MET. Additionally, two LGG patients were excluded due to radiological progression after obtaining histological sample, i.e. four patients were excluded in total.

Additionally, subjects 5, 6, 7, and 19 were excluded for external reviewer analysis due to known tumor type by the reviewers. Subjects 12 and 16 were excluded for external reviewer analysis due to progression after obtained histopathological analysis (Table 5).

This resulted in a total final cohort of 22 (2 MET and 2 LGG with progression excluded) patients, albeit only 20 (4 à priori known tumor diagnoses by readers and 2 LGG with progression excluded) for the external neuroradiological reviewer assessment, which were included in the analysis concerning the radiological value of adding the APTw images (Table 5).

### Statistical analysis

Normality testing was performed with boxplots, outliers, skewness, kurtosis and a Shapiro–Wilk’s test. Mann-Whitney U was chosen for analysis of mean, max, min and range APTw signal measured in Gd enhanced or hypointense/hyperintense zones identified as lesional tissue on conventional MRI for the HGG and LGG groups. Status of promoter region methylation of O6-methylguanine DNA methyltransferase (MGMT) and Isocitrate dehydrogenase 1 and 2 (IDH 1/2) mutations were also assessed. A logistic regression model was constructed for LGG and HGG and Receiver Operating Characteristic (ROC) curves were assessed and area under the curve (AUC), sensitivity and specificity were calculated, with significance level set at P-value < .05, [35]. Statistical analysis was performed with SPSS® v. 24.0 (IBM Corp., New York, NY, USA; formerly SPSS Inc., Chicago, IL, USA).

## Results

**Fig 3:**
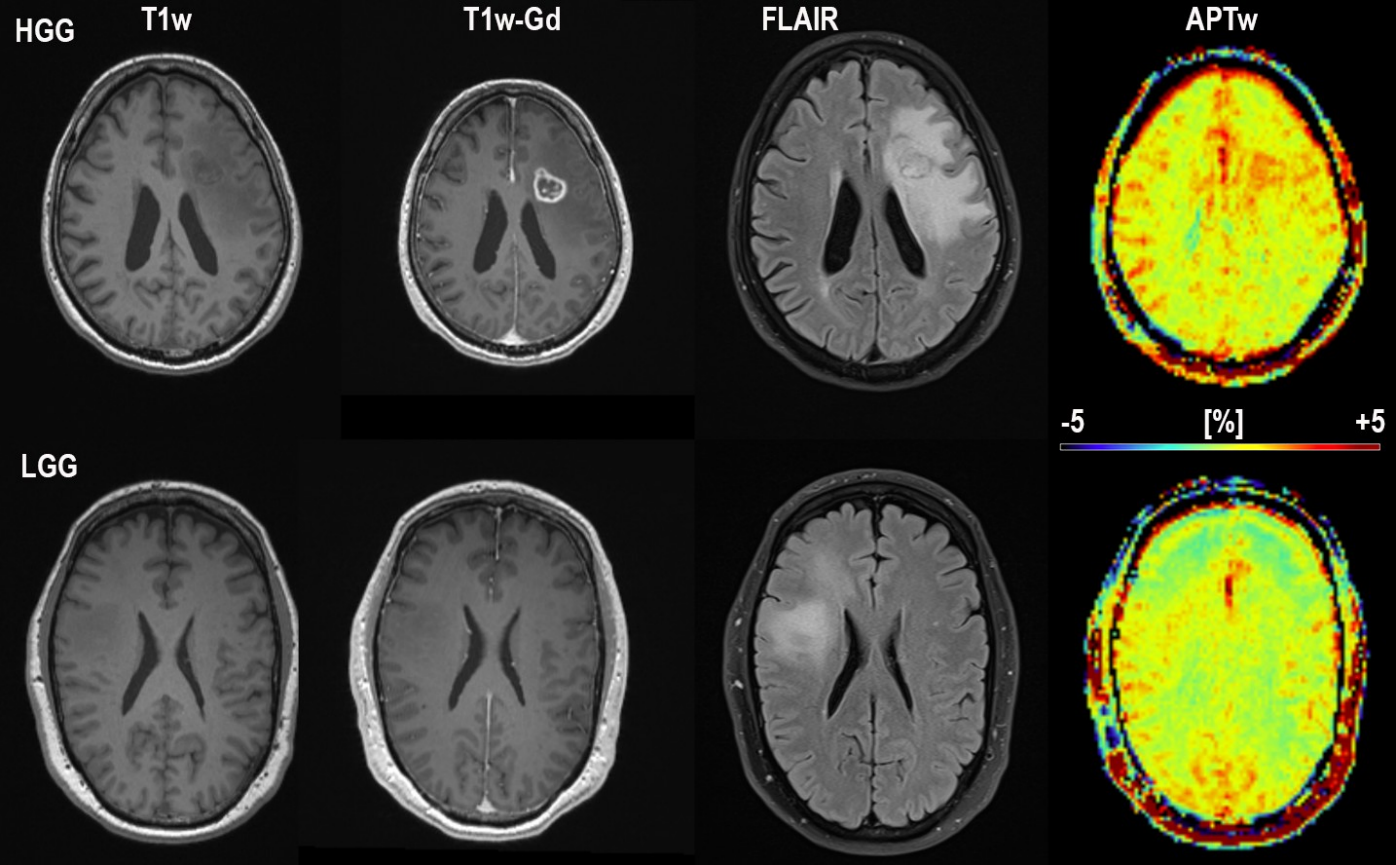
**Conventional MRI; T1-MPRAGE without contrast, T1-MPRAGE with Gadolinium, FLAIR and APTw in color for subject one (HGG; top) and subject 3 (LGG; bottom) The APTw signal intensity for the high grade glioma subject at the top is distinctly higher than for the low grade glioma subject at the bottom.** Also notice the increased APTw signal in blood vessel regions. Subject 1 HGG (misdiagnosed by reader 1 and 3 as MET, correctly diagnosed by APTw) and subject 3 LGG (correctly diagnosed by all)”.

**Fig 4:**
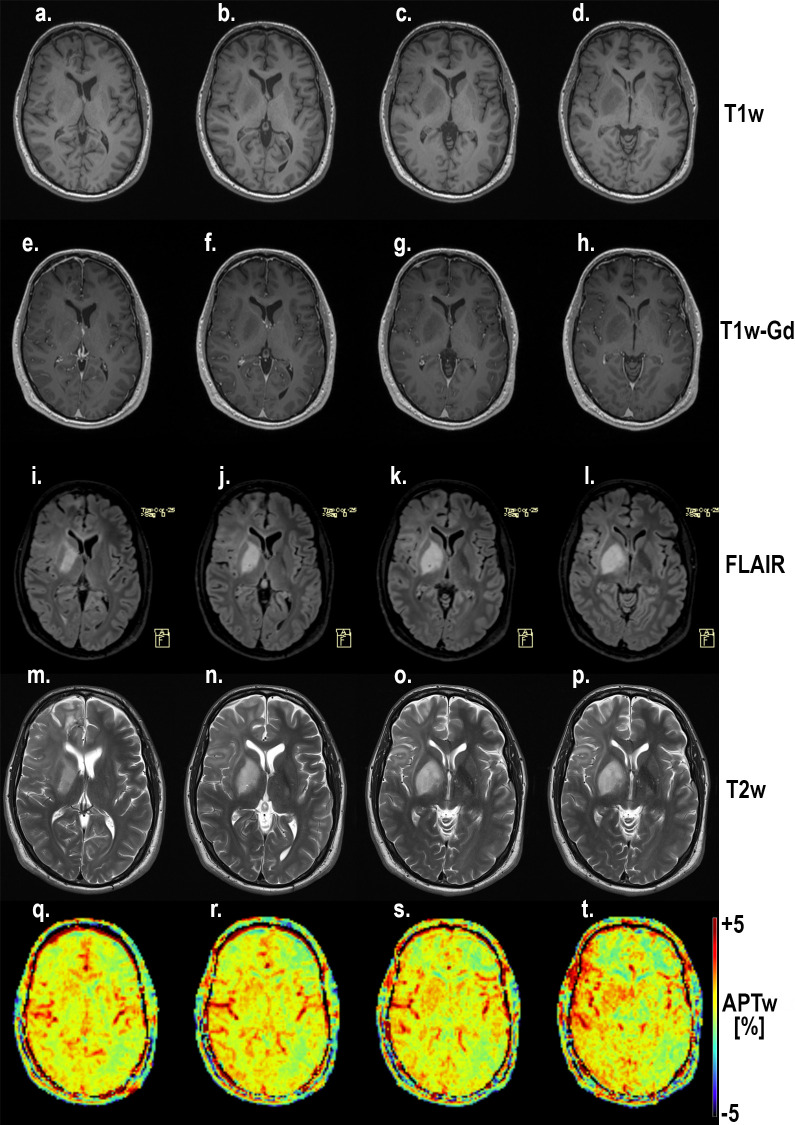
Four slices through the lesion of subject 5 with a high grade glioma (HGG). Notice the coherence between the increasing hypointensity on T1-MPRAGE (a-d), hyperintensity on FLAIR (e-h), increased hyperintensity on T2w MRI (i-l) and increased APTw signal (m-p). Also notice the increased APTw signal in blood vessel regions. Gd-enhancement was studied with T1w; T1-MPRAGE. Hyperintensities on T2 FLAIR and T2 turbo spin-echo of conventional pre-contrast protocols were correlated to T1w-Gd enhancement where possible, otherwise hypointensity.

**Fig 5:**
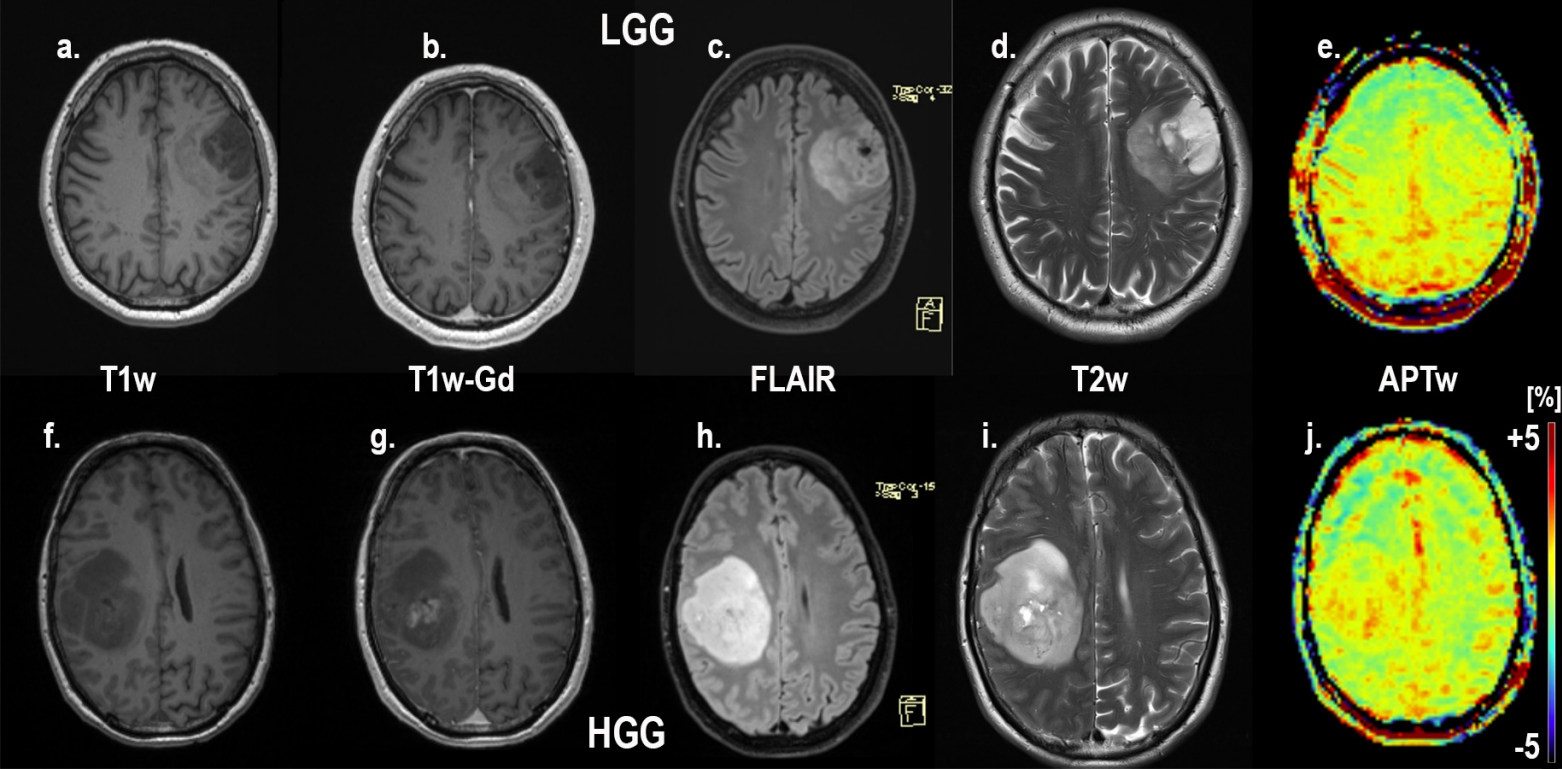
T1-MPRAGE without contrast, T1-MPRAGE with Gadolinium, FLAIR, T2w and APTw in subject 9 with a low grade glioma (a-e) and subject 10 with a high grade glioma (f-j). Of note is the increased APTw signal in the high grade glioma (j) compared to the low grade glioma (e). on APTw. Also notice the increased APTw signal in blood vessel regions of all patients. Subject 9 LGG (correctly diagnosed by all) and subject 10 HGG (misdiagnosed by the initial radiological classification as MET, correctly diagnosed by APTw).

**Fig 6:**
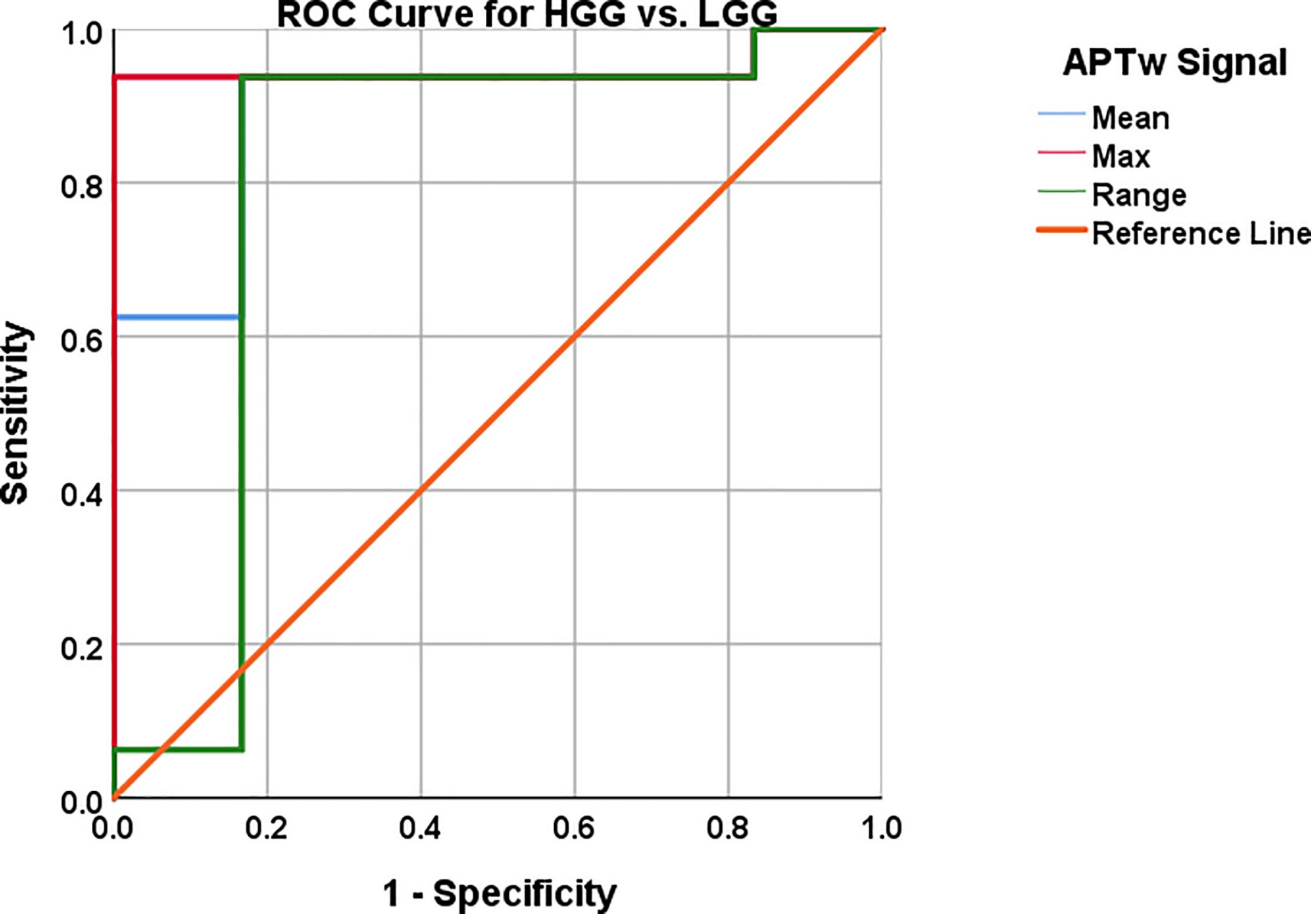
Z-spectra and magnetization transfer ratio asymmetry spectra for subjects; subject 13 (HGG) and 9 (LGG) within tumor and in contralateral normal appearing white matter.

An example of the difference in APTw signal between MET, LGG and HGG is shown in Figs 1–3; HGG (Subject 18, Figs 1 and 2, subject one, Fig 3) showing higher intensities within the lesion compared to the LGG (subject 17, Figs 1 and 2, subject three, Fig 3). This assessment corresponded with that from Gd-enhancement on T1w-Gd enhanced imaging (Fig 3, Table 5, subject one demonstrated tumour enhancement, subject three did not demonstrate any tumour enhancement). Fig 4 shows another illustration of increases in APTw signal overlapping anatomically with hypointensity on T1w MRI, hyperintensity on FLAIR and T2w MRI. Summarized in Table 5, there was only mixed iso and hypointense signal on Gd-enhanced T1-MPRAGE (Table 5), hypointensity on T1-MPRAGE, and hyperintensity on FLAIR overlapped with increasing APTw signal (Fig 4). A final illustration in Fig 5 shows that a patient with LGG (subject 9) has less APTw signal enhancement within lesional tissue when compared to a patient with HGG (subject 10). Of note also is the increased APTw signal in the blood vessels, here being especially clear in highly vascular areas within sulci (Figs 3–5). Additionally, paired examples of Magnetization Transfer Ratio asymmetry spectra as well as Z-spectra for a HGG (Subject 13, Table 1) and a LGG (Subject nine, Table 1) are shown in Fig 6, confirming the increased asymmetry for the high grade glioma. As mentioned before, due to the low number of single metastasis in this material no statistical evaluation was performed with respect to high grade glioma compared to metastasis. Subject 25 and 26, both adenocarcinoma with gastrointestinal origin exhibited APTw mean, max and min percent signals of; 2.25, 2.77, 1.31 and 2.72, 3.19 and 2.21, respectively (Table 1). The metastases had higher APTw max signal than all included LGGs but similar mean max and min APTw signal to HGGs, rendering the two metastases indistinguishable from HGG, (Table 1).

**Table 1:** Patient demographics, histopathology with corresponding APTw signal intensity and gene mutation status.

Patient/Sex/Age at diagnosis	Gene mutation status	Histopathology	APTw Mean (%)	APTw Max (%)	APTw Min (%)
1/M/63	IDHwt, MGMT meth	GB grade 4	2.37	2.61	2.08
2/F/51	IDHwt, unknown MGMT status	GB grade 4	3.43	3.89	2.90
3/M/48	IDHwt, unknown MGMT status	Astrocytoma grade 2	1.72	1.98	1.09
4/M/57	IDHwt, unknown MGMT status	GB grade 4	2.09	3.00	0.83
5/M/43	IDH mut, MGMT meth	GB grade 4	2.30	2.88	1.75
6/M/72	IDHwt, MGMT meth	GB grade 4	2.33	2.77	1.41
7/M/52	IDHwt, unknown MGMT status	GB grade 4	0.92	1.48	0.14
8/M/30	IDH mut, unknown MGMT status	GB grade 4	5.08	5.83	3.93
9/M/57	IDH mut, 1p19q-codeletion, unknown MGMT status	Oligodendroglioma grade 2	0.88	1.21	0.41
10/M/52	IDH mut, MGMT meth	GB grade 4	2.25	2.88	1.61
11/M/55	IDH status unknown, 1p19q-codeletion, MGMT unknown	Oligodendroglioma grade 2	2.20	2.35	1.93
12/M/53	IDH mut, unknown MGMT status	Oligoastrocytoma grade 2	1.74***	2.23***	1.36***
13/M/73	IDHwt, MGMT meth	GB grade 4	4.24	4.86	2.79
14/M/43	IDH mut, unknown MGMT status	Diffuse astrocytoma grade 2	1.68	2.06	1.27
15/F/53	IDHwt, GFAP+, MGMT non meth	GB grade 4	2.07	3.08	1.25
16/M/46	IDH and MGMT status unknown	Oligodendroglioma grade 2	2.28***	2.86***	1.54***
17/M/62	IDH mut, GFAP+, no 1p19q-codeletion, deletion within 19q, MGMT meth	Diffuse astrocytoma grade 2	0.94	2.11	0.02
18/M/31	IDH mut, unknown MGMT status	GB grade 4	2.98	3.80	2.35
19/M/63	IDHwt, MGMT meth	GB grade 4	2.16	2.62	1.07
20/M/61	IDHwt, MGMT meth	GB grade 4	2.07	2.65	1.65
21/F/68	IDHwt, MGMT meth	GB grade 4	2.29	2.98	1.57
22/F/71	IDH status unknown, MGMT meth	GB grade 4	2.80	3.21	2.28
23/M/26	IDHwt, MGMT non meth	GB grade 4	2.14	3.12	1.40
24/M/48	IDHwt, MGMT non meth	Diffuse Astrocytoma grade 2	1.54	1.96	1.34
25/M/65	****	Adenocarcinoma—GI origin	2.25	2.77	1.31
26/M/76	****	Adenocarcinoma—GI origin	2.72	3.19	2.21

Note

*** Excluded for further analysis due to radiological progression after obtaining histological sample

**** Metastatic adenocarcinoma–excluded, M = Male, F = Female, IDHwt = IDH wildtype, IDHmut = IDH mutated, GB = Glioblastoma, MGMT met = MGMT promoter methylated.

### Quantification of APTw

The final cohort (a subset of Table 1) used for further analyses consisted of 18 males and 4 females; 16 HGG (Glioblastoma grade 4; n = 15, 1 Anaplastic Astrocytoma grade 3), 6 LGG (Astrocytoma grade 2 (n = 1), Oligodendroglioma grade 2 (n = 2) and Diffuse Astrocytoma grade 2 (n = 3)). LGG were composed solely of males in this cohort. Mean age at diagnosis was 52 years (LGG) and 54 years (HGG) with range being 26–73 years for HGG and 43–62 years for LGG. As mentioned in the Materials and Methods section two patients with LGG were excluded due to radiological progression after obtaining histological samples and two MET patients with adenocarcinoma from GI origin were excluded due to insufficient number of metastasis subjects for statistical analysis.

The APTw image values in both LGGs and HGGs were elevated and found to differ between the two groups. The mean APTw signal integral in HGG (n = 16) was higher (2.60±.97%) than in LGG (n = 6) (1.49±.50%), P-value = .005 (Tables 2 and 3). Furthermore, the HGG exhibited a broader range of mean APTw signal integral (.92–5.08) than the LGG (.88–2.20) (Table 2). Maximum, minimum and range (i.e. within subject max APTw–min APTw) of APTw integrals were higher in the HGGs than in the LGGs; 3.23±1.00% vs. 1.95±.39%, 1.81±.91% vs. 1.01±.69% and 1.42±.45% vs. .94±.59%, respectively (P–values < .002; .055; .032) (Tables 1–3).

**Table 2:** APTw signal mean, max, min and range for HGG, LGG and MET.

	Group		APTw (%)
APTw-mean	HGG	Average	2.60
		Minimum	0.92
		Maximum	5.08
		Range	4.16
	LGG	Average	1.49
		Minimum	0.88
		Maximum	2.20
		Range	1.32
	MET	Average	2.49
		Minimum	2.25
		Maximum	2.72
		Range	0.47
APTw-max	HGG	Average	3.23
		Minimum	1.48
		Maximum	5.83
		Range	4.35
	LGG	Average	1.95
		Minimum	1.21
		Maximum	2.35
		Range	1.14
	MET	Average	2.98
		Minimum	2.77
		Maximum	3.19
		Range	0.42
APTw-min	HGG	Average	1.81
		Minimum	0.14
		Maximum	3.93
		Range	3.79
	LGG	Average	1.01
		Minimum	0.02
		Maximum	1.93
		Range	1.91
	MET	Average	1.76
		Minimum	1.31
		Maximum	2.21
		Range	0.90
APTw-range	HGG	Average	1.42
	LGG	Average	0.94
	MET	Average	1.22

Note: HGG (n = 16); LGG (n = 6); MET (n = 2).

**Table 3:** Mann-Whitney U test for distinguishing low grade glioma and high grade glioma using APTw signal.

	APTw image mean	APTw image max	APTw image min	APTw image range
Mann-Whitney U value	9.0	5.0	22.0	19.0
P-value =	**.005**	**.002**	.055	**.032**

Note: HGG (n = 16), LGG (n = 6), outcome of statistical test.

For the Mann-Whitney U test, HGG (n = 16) exhibited mean ranks of APTw signal intensity mean (13.88), max (14.19), min (13.13) and range (13.31). LGG (n = 6) had mean ranks for mean, max, min and range APTw; (5.17), (4.33), (7.17) and (6.67) (Table 3), respectively.

As the number of metastasis (MET) were only 2 in the final cohort, no further analysis of comparison between MET and HGG or LGG was performed. However, from Table 2 it can be noted that the group average for APTw mean, max and min signal in MET was lower than in HGG and higher than in LGG (Table 2).

### Results from S1 File

HGG were shown to exhibit the highest signal for Mean and Max and Range APTw %; 2.28, 3.18, 1.82, respectively (S1 Table). APTw Mean and Max signal were found to be significant predictors by Mann Whitney U testing of LGG and HGG, p-values = .018 and .005, respectively (S3 Table).

ROC-analysis for distinction between HGG and LGG showed comparable cut off values and performance when compared to initial 10 pixel ROI ROC-analysis (S4 and S5 Tables, S1 Fig). The logistic regression model with combined Mean and Max APTw signal showed identical AUC of .896 for the combined parameters and Max APTw signal (S4 Table). Max APTw signal intensity yielded highest Sensitivity with 93.8% while the combined signal intensity model showed the best specificity; 100% (S4 Table).

The classification by the logistic regression models based on 10 Pixel ROI and whole-tumor ROI misclassified three patients each as seen in S5 Table. The model based on the 10 Pixel ROI segmentation misclassified subject 7 as a HGG, subjects 3 and 17 as LGG (S5 Table). The model based on whole tumor slice segmentation also misclassified subject 7 as HGG and subjects 11 and 24 as LGG (S5 Table).

### ROC analysis of single biomarkers and combined biomarkers

**Fig 7:**
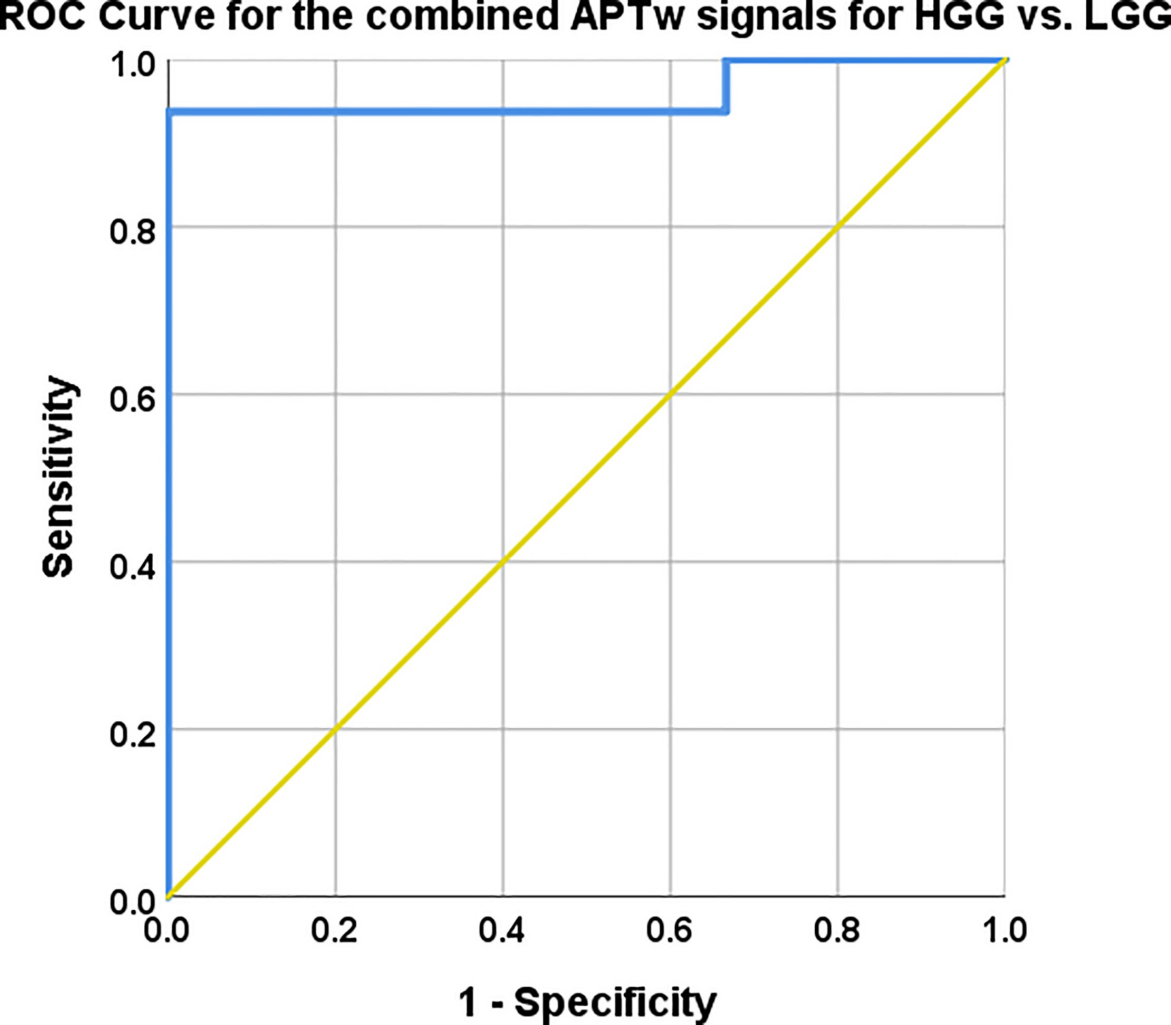
AUC, 95% CI, sensitivity and specificity with cut off values reported in Table 4.

Cut off values were chosen based on highest possible sensitivity and specificity as the clinical motivation (time to treatment, prognosis, survival) for this infers radiologists need to both diagnose high grade glioma and low grade glioma with a high probability. Best suitable cut off values in the individual ROC curve analyses for mean, max and range APTw image intensity were 1.90%; 2.48% and .91%, respectively (Table 4, Fig 7). Sensitivity and specificity were 93.8% and 83.3%, 93.8% and 100% as well 93.8% and 83.3% for mean, max and range of APTw signals, P-value = .005; .002; .033, respectively (Table 4, Fig 7). For single biomarkers; Mean and max APTw exhibited the highest AUC, .896 and .948, respectively (Table 4, Fig 7).

**Table 4:** ROC analysis for distinguishing HGG and LGG using APTw signal intensity.

APTw	Area under the curve (AUC)	P-value =	95% Confidence Interval		Cutoff/ Sensitivity/ Specificity
			Lower Bound	Upper Bound	
Mean	.896	.005	.751	1.00	1.90% / 93.8% / 83.3%
Max	.948	.002	.846	1.00	2.48% / 93.8% / 100%
Range	.802	.033	.523	1.00	.91% / 93.8% / 83.3%
Combined*	.958	.001	.873	1.00	.38** / 93.8% / 100%

Note: Corresponding table for Figs 3 and 4. Showing AUC, APTw signal cut off (%), sensitivity (%), specificity (%) with 95% CI and P-values.

* Mean, max, range combined.

** Probabilistic cutoff value.

The constructed logistic regression model exhibited P-values < .002 for Omnibus tests of model coefficients, Nagelkerke R^2^ of .722, Hosmer-Lemeshow Test of .902.

**Fig 8:**
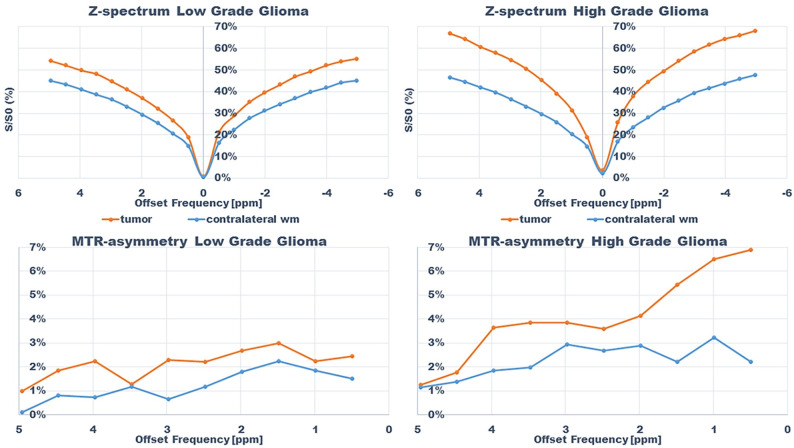
Mean, max and range APTw signal combined with logistic regression. AUC, 95% CI, Sensitivity and specificity with cut off values reported in Table 4. The combined model mislabelled subjects 3 and 17 as they were labelled HGG in the model but are histologically verified LGG, also subject 7 was mislabelled as a LGG whereas it is histologically a Glioblastoma, Table 1.

The combination of mean, max and range of APTw signal yielded 93.8% sensitivity and 100% specificity with a probabilistic cut of value 0.38, for the distinction between LGG and HGG (Fig 8). The corresponding AUC was .958, 95% CI .873–1.00, P-value = .001.

The predictive capacity for LGG vs. HGG of each APTw signal; mean, max and range differed between the chosen APTw signal parameters as shown in (Fig 7). The Logistic Regression with combined APTw; mean, max and range depicted in (Fig 8) showed similar predictive capacity as max APTw signal the combined approach yielded a greater AUC (.958 vs. .948) and higher Confidence Interval (.873–1.00 vs .846–1.00) (Figs 7 and 8, Table 4).

### Radiological diagnosis

All 26 patients presented with supratentorial lesions: 9 were in the left hemisphere, the rest in the right hemisphere. FLAIR, T2w imaging, pre- and post-contrast T1w imaging was used for radiological diagnosis by readers. In our study and recent studies by others [30,36,37], LGGs were shown to also have a non-zero APTw signal, which our method also clearly shows by co-utilizing T1w-Gd+T2w+FLAIR in the placement of ROIs on APTw MRI. Even though the APT signal is not deemed high in all LGG, it is elevated as described in (Tables 1 and 2). Furthermore, all subjects’ lesions displayed hyperintensity on FLAIR MRI, also illustrated in (Figs 3–5).

The primary radiological diagnosis pre-operatively was metastasis, high grade and low-grade tumor with n = 6, n = 8 and n = 12, respectively. As four (2 MET and 2 LGG, subjects 12, 16, 25 and 26) of the initial 26 patients were excluded, the final cohort evaluated consisted of 22 patients. Retrospective revision of the initial radiological classification of these 22 patients demonstrated that, 8 patients (4 HGG as LGG, and four HGG as MET) were initially incorrectly classified (Table 5). Follow-up of the initial exam; the three reviewers mislabeled 4, 4 and 7 patients, respectively, out of a total of 20 patients ultimately assessed in the second review by the three independent neuroradiologists (Table 5). In this follow up review, a total of six patients from the initial cohort of 26 patients were excluded for the readers’ assessment: four subjects due to known disease to readers. We decided, for the second review by the neuroradiologists, to exclude an additional two patients as the histopathological diagnosis was made a few years before the APTw imaging examination and therefore we cannot exclude radiologically non-stable disease according to RANO-criteria in the interval. (Table 5). The external readers, as seen from Tables 1 and 5, most notably mislabeled one HGG as LGG and one LGG as HGG (reader 1). Reader 2 mislabeled two LGG as HGG and reader 3 mislabeled two LGG as HGG while mistaking one HGG for a LGG, corresponding to 80%, 80% and 65% correct diagnoses for readers 1, 2 and 3 respectively, (Table 5).

**Table 5:** Classification of brain lesions into HGG/LGG/MET at primary presentation of disease and follow-up review with 3 readers and quantified intralesional mean APTw signal with cut off >+2.0% and qualitative assessment of T1w Gd enhancement.

Subject/Initial radiological classification	Reader 1	Reader 2	Reader 3	Classification with APTw-mean > +2.0%	Presence of T1w-Gd enhancement
1/Correct HGG	Incorrect (MET)*	Correct	Incorrect (MET)*	Correct	Yes, ring enhancing lesion
2/Correct HGG	Correct	Correct	Correct	Correct	Yes, three smaller lesions
3/Correct LGG	Correct	Correct	Correct	Correct	No, mainly hypointense lesion
4/Incorrect (LGG)* HGG	Correct	Correct	Correct	Correct	No, mixed iso and hypointense lesion
5/Correct HGG	**	**	**	Correct	No, mixed iso and hypointense lesion
6/Incorrect (MET)* HGG	**	**	**	Correct	Yes, ring enhancing lesion
7/Correct HGG	**	**	**	Incorrect (LGG)*****	Yes, three large ring enhancing lesions
8/Correct HGG	Correct	Correct	Correct	Correct	Yes, enhancing ring lesion
9/Correct LGG	Correct	Correct	Correct	Correct	No, mixed iso and hypointense lesion
10/Incorrect (MET)* HGG	Correct	Correct	Correct	Correct	Yes, multiple ring enhancing lesions
11/Correct LGG	Incorrect (HGG)*	Incorrect (HGG)*	Incorrect (HGG)*	Incorrect (HGG)****	Yes, variably enhancing lesion
12/Correct LGG	***	***	***	***	Yes, mixed iso and hypointense areas with variable enhancement
13/Incorrect (MET)* HGG	Incorrect (MET)*	Correct	Correct	Correct	Yes, ring enhancing lesion
14/Correct LGG	Correct	Incorrect (HGG)*	Incorrect (HGG)*	Correct	Yes, multiple ring enhancing lesions, mixed hypo and isointense areas
15/Incorrect (LGG)* HGG	Correct	Correct	Correct	Correct	Yes, hypointense, minimal variable enhancement in lesion
16/Correct LGG	***	***	***	***	Yes, mainly hypointense areas with some minimal variable enhancement
17/Correct LGG	Correct	Correct	Correct	Correct	No, hypointense lesion
18/Incorrect (LGG)* HGG	Correct	Correct	Correct	Correct	Yes, multiple enhancing lesions
19/Correct HGG	**	**	**	Correct	Yes, three ring enhancing lesions
20/Correct HGG	Correct	Incorrect (MET)*	Incorrect (MET)*	Correct	Yes, smaller ring enhancing lesion
21/Correct HGG	Correct	Correct	Correct	Correct	Yes, ring enhancing lesion
22/Incorrect (MET)* HGG	Correct	Correct	Correct	Correct	Yes, ring enhancing lesion with additional minor adjacent enhancement
23/Incorrect (LGG)* HGG	Incorrect (LGG)*	Correct	Incorrect (LGG)*	Correct	No, mixed iso and hypointense lesions
24/Correct LGG	Correct	Correct	Correct	Correct	No, hypointense lesion
25/Correct MET	Correct	Incorrect (HGG)*	Incorrect (HGG)*	Excluded	Yes, ring enhancing lesion
26/Correct MET	Correct	Correct	Incorrect (HGG)*	Excluded	Yes, cystic lesion, complete enhancement of spherical cyst

Note

* Incorrect classification with misdiagnosis within parenthesis

** Were not assessed by reviewers due to known tumor type

*** Excluded due to radiological progression after obtaining histological sample

**** APTw-Mean of > +2.0% enabled correct diagnosis

*****APTw-Mean of <+2.0% did not enable correct diagnosis, HGG = High grade glioma, LGG = Low Grade Glioma, MET = Metastasis. All subjects’ lesions displayed hyperintensity on FLAIR sequence.

By utilizing a cutoff of 2.0% average of the max APTw image intensity in the preoperative assessment, all mislabeled LGG could be rectified to HGG non-invasively with APTw imaging (Table 5). APTw maximum signal >2.48% correctly diagnosed 21 out of 22 patients, correcting all but with one misdiagnosed, subject seven (Table 5).

Histopathological diagnosis confirmed that four of the radiologically diagnosed LGGs during the initial radiological classification would have been diagnosed as glioblastoma by utilization of either mean APTw > 2.0% or max APTw signal of > 2.48% (see Tables 1 and 5).

### MGMT methylation status, IDH mutation status

Only 9 out of 16 HGG patients exhibited MGMT methylation: subjects 1, 5, 6, 10, 13, 19, 20, 21, 22 (all histologically confirmed glioblastoma) (Table 1). Five of the 16 HGG were not tested for MGMT methylation. Two patients (2 HGGs) that were assessed for MGMT promoter methylation were found to be non-methylated; subjects 15 (Glioblastoma grade 4) and 23 (Anaplastic Astrocytoma grade 3). Note, subject 24, histological LGG, was also analyzed and found to be MGMT promoter non-methylated while subject 17, also a LGG, was found to be MGMT promoter methylated, in total 10 patients had MGMT promoter methylation (9 HGGs and 1 LGG) (Table 1). For LGG and HGG, 7 out of 22 patients (4 glioblastoma, 3 LGG) exhibited either or both IDH 1 and 2 mutations, i.e. subjects 5, 8, 9, 10, 14, 17 and 18 (2 LGGs, cases 12 and 16 were excluded) (Table 1). 13 subjects had glioma with IDH wildtype with APT mean 2.26% vs 2.30% for IDH mutant (Table 1).

Mean, maximum, minimum, and range of APTw intensities could not distinguish HGG with or without MGMT methylation (9 glioblastomas with MGMT promoter methylation vs. 2 HGGs (Glioblastoma Grade 4 = 1, Anaplastic Astrocytoma Grade 3 = 1 without MGMT methylation), 5 glioblastoma were excluded as MGMT analysis was not done), P-values > .05 for all APTw image intensity variables, or mathematically insufficient number for statistical analysis. IDH status in terms of IDH 1 or 2 mutation for LGG (3 LGGs with mutation vs. 2 LGGs without) was, due to an individual group size < 4, not enough for statistical analysis. Distinction between IDH 1/2 mutated lesions with LGG and HGG (n = 13 wildtype, n = 7 mutated) pooled together was non-significant with P-values > .05 for all APTw variables.

Mean APTw image intensity in the complete glioma cohort (glioma without MGMT methylation; n = 3 (1 histopathological proven LGG grade 2 and 1 Glioblastoma grade 4, 1 Anaplastic Astrocytoma Grade 3)) and glioma with MGMT methylation; n = 10 (nine histopathological glioblastoma grade 4, one LGG with diffuse Astrocytoma Grade 2)) were of insufficient number for statistical analysis. Gliomas with evident MGMT methylation had a higher mean APTw = 2.38±.81% (n = 10, subjects 1, 5, 6, 10, 13, 17, 19, 20, 21 and 22) compared to those without MGMT methylation; mean APTw = 1.92±.33% (n = 3 subjects).

## Discussion

Our primary aim of this study was to assess the radiological and clinical value of using APTw imaging in the initial clinical pre-surgical assessment of primary brain tumors and to distinguish between brain tumors of low versus high-grade (LGG versus HGG), which might influence surgical planning and follow-up treatment. The results demonstrate that an assessment including APTw images corresponds better to histopathology than the radiological assessment without the APTw images (Table 1). This is of clinical importance as the management and treatment for HGG versus LGG gliomas are in many cases fundamentally different. Our study demonstrates that adding APTw images to the initial radiological assessments greatly improved pre-surgical diagnosis (Table 5). Furthermore the finding of higher APTw signal in HGGs versus LGGs (Tables 2 and 4) in this study is in congruence with earlier research studies where ATPw imaging has shown promise to noninvasively differentiate LGGs from HGGs [25,28,32,38,39].

We show here that utilization of either mean or max APTw signal outperformed or equaled the original reading on initial presentation as well as the follow-up with the three experienced neuroradiologists at our institution (Table 5). Of these two assessment approaches, max APTw signal was somewhat more accurate with 100% specificity vs. 83.3% for mean APTw signal while being equally sensitive: 93.8% (Table 1, Tables 4 and 5, Figs 7 and 8). Furthermore, max APTw signal showed higher AUC of .948 versus .948 and CI of .846–1.00 versus .751–1.00 for mean APTw signal (Table 4). The logistic regression model utilizing max, mean and range APTw signal misclassified subjects 3 and 17 as HGGs, it also misclassified subject 7 as a LGG whereas it is a HGG (Fig 8).

Adding APTw imaging in the radiological review improved correct diagnosis by distinguishing 4 lesions tentatively diagnosed as LGGs, as glioblastomas, subsequently histopathologically verified (Tables 1 and 5).

There are previous studies evaluating the APTw signal in defined cohorts of HGG and LGG tumors [39]. However, none of these previous studies mentioned in the systematic review [39] have evaluated the complementary diagnostic value of APTw imaging to the radiologists’ assessment of brain tumors in a prospective manner and in clinical routine for pre-surgical assessment to improve accuracy of diagnosis.

A previous study has demonstrated that APTw signal increases from low to high grade [38]. Additionally, the same study demonstrated that APTw image intensity in the lesion could successfully distinguish grade 2 from 3 and grades 2 and 3 from 4 [38], which is in congruence with our study where LGGs (all subjects WHO grade 2) had a mean APTw signal integral of 1.49%, whereas the HGGs (all subjects WHO grade 4 glioblastoma) had a mean of 2.65% (Table 1). APTw MRI has been shown to be a better predictor of tumor grade than the apparent diffusion constant (ADC) and dynamic perfusion derived cerebral blood flow (CBF) [38], with sensitivity and specificity reaching 95% for distinction between grade 2 and 4 gliomas [38]. This has also been demonstrated Paech et al. [36].

Accordingly, our study reached 93.8% in sensitivity and 100% in specificity for max APTw image intensity (Table 4). For distinction of grade 2 from grade 4 glioma with APTw MRI, Bai et al. [38] have reported an AUC of .997 and 95% CI .89–1.00. However, they did not specify sensitivity and specificity, but provided ROC-curves which show sensitivity and specificity >90% but less than 100% for distinction between grade 2 and grade 4 glioma [38]. Max APTw signal yielded 100% specificity which also utilisation of mean, max and range with logistic regression resulted in where only 3 subjects where mislabeled; 2 LGGs as HGGs and one HGG as a LGG..These findings are comparable to those of both Bai et al. [38] and Jiang et al. [32].

Similar to our study, higher APTw image intensity has been observed for tumor vs. normal tissue in animal glioma models [19], and more recently for distinction between benign and atypical meningiomas [40]. Additionally, APTw image intensity has previously been shown to correlate well with higher cellularity and proliferation when assessing brain tumor tissue [32,38]. An older study with both CT and MRI showed a 50% false positive rate when comparing neuroradiological assessment with histological diagnosis [5]. Similarly, our study showed that the radiological tentative routine clinical pre-operative diagnosis only had 18 out of 26 patients correctly diagnosed (69%) when evaluating conventional MR images (FLAIR, T2w, and pre- and post- Gd T1w images) solely (Table 5). However, using a max APTw cutoff > 2.48% showed greater accuracy, correctly diagnosing 21 out of 22 (96%) patients (Tables 1 and 5).

In this present study, focusing on the feasibility of routine clinical use of APTw, we found that the mean APTw signal could effectively distinguish between LGG and HGG. The higher mean or max APTw in HGG both altered the pre-operative radiological diagnoses in four subjects classified as LGG, as these were in fact glioblastoma; verified with histopathological analysis (Table 1). Speculatively, the higher mean and max APTw we found in HGG (Table 2) may be attributed to overexpressed proteins such as peroxidoxin 1 and 6, transcription factor BFT3, alpha B crystallin, hemoglobin and albumin as previously investigated [41].

In our cohort, the MGMT promoter methylated gliomas (n = 10) had a higher overall mean APTw signal intensity of 2.38% compared to 1.92% for non-methylated gliomas (n = 3), albeit, the differences in levels of APTw signals could not be statistically analysed due to insufficient number of patients (Table 1). Nonetheless, we show that in patients with suspected glioma (HGG and LGG); mean APTw signal is more elevated in MGMT methylated patients 2.38% (n = 10) vs. 1.92% (n = 3). This is in conjunction with a previous study that has shown the APTw signal values (mean, variance, 50^th^ percentile, 90^th^ percentile and width (10–90)) to be higher in glioblastomas with a methylated MGMT promoter than in those without [42]. Furthermore, recent work has also shown that it is possible to detect IDH mutant grade 2 gliomas by utilization of APTw MRI [36,37]. The evaluation of APTw images between IDH 1/2 mutated LGG and wildtype LGG (n = 3 with mutation and 2 without) could not be performed in our cohort due to lack of statistical power (individual group size < 4).

The use of maximum, minimum and mean values of APTw imaging in glial tumors has been shown to discriminate between low and high grade tumors [32,43]. In our study, the mean APTw signal was 74% higher in HGG (2.60% / 1.49%) and the max APTw signal was 66% higher in HGG vs. LGG (3.23% / 1.95%) (Table 2). This is in accordance with previous findings demonstrating that proliferation and increasing cellularity within tumor tissue is associated with higher APTw signal [32]. In similarity with our study (Table 2), these investigators found that average mean and max APTw signal increased with higher WHO grade in glioma with HGG grade 4 having 34% (2.43%/ 1.82%) higher mean APTw signal than LGG grade 2 and 64% higher max APTw signal (3.39% / 2.07%). Our findings of AUCs ranging from .802 to .948 for mean, max and range of APTw signal with sensitivities 93.8–93.8% and specificities 83.3–100%, respectively (Table 4), are quite similar to Sakata et al. [44] with their sensitivity and specificity ranging from 66.7–83.3% and 75–100%, respectively. Additionally, APTw MRI and ADC analyses have been shown, when used in concordance, to help differentiate between LGG and HGG, AUC = .91 [27]. However, our approach with logistic regression has the advantage of 93.8% sensitivity, 100% specificity and AUC .958 (Fig 8).

Not all genes and proteins become upregulated; some can also be downregulated. Proteins vary in size (amount of NH groups [44]) and mobility, thus varying the accessibility of the amide protons, which may also be the cause of why some of our patients exhibited, relative to the other subjects, low mean APTw signal values (Table 1). As we are looking at a single signal intensity, combining mobile protein CEST, pH, and some conventional semi-solid magnetization transfer effects, it is virtually impossible to identify the proteins that contribute to the APTw signal changes. Gliomas of grades 2, 3 and 4 have been shown to have different types of upregulated proteins: out of 650 membrane proteins, only 10, 3 and 46 were congruent across grades 2, 3 and 4 [45]. When comparing total protein content for grades 2, 3 and 4, grade 4 had the highest content with increasing total content as grade increases [45]. Interestingly, some specimens of grade 2 and 3 showed marginally higher total protein content, which corresponds to our findings of relatively low mean APTw image intensity in one glioblastoma case of .92 (subject seven), extremely low in a LGG of .88 (subject nine) and exceedingly high APTw image intensity for one LGG of 2.20 (subject 11, Table 1). In terms of unique proteins; grades 2, 3 and 4 exhibited 177, 120 and 594 differing proteins along with the correlated increase in grade and protein content in gliomas [45]. Even though it is unclear which subset of proteins the APTw MRI actually detects, it is clear that APTw MRI is well suited as a complement to the initial clinical radiological differentiation of LGG from HGG.

### Limitations

Our study has some limitations, one being the relatively small cohort size.

Nevertheless, it has been shown that in order to have a mathematically justifiable possibility of rejecting the null hypothesis, the minimum sample size for group comparisons with an alpha = .05, needs to have a minimum of n = 4 for each group [46,47]. Mann-Whitney U test is superior to student-t test when sample sizes are small and unequal and normality assumptions are violated [48]. The Shapiro-Wilks test has a tendency to show that data is normally distributed when sample sizes are small. For our data it showed mean and maximum APTw signal values that were not normally distributed (< .05), while minimum and a range of APTw signal values (> .05) followed normal distribution. This suggests that the group size was of adequate number of subjects as the test managed to detect non-normal distributions in the data [49]. In addition, the Shapiro Wilk’s test has a minimum threshold of 3 subjects for testing of normality, while we had a minimum of 6 in our cohort [50]. An additional limitation is that the utilization of APTw imaging did not confer perfect discrimination as subject seven was misdiagnosed as a low grade tumor whilst it was a glioblastoma (Table 5).

Our chosen approach with a 10 pixel ROI may be difficult to reproduce and in order to reduce risk of bias we have added an additional analysis of the material based on one whole tumor ROI (excluding cystic/necrotic/hemorrhagic areas) selected on T1w-Gd / T2w FLAIR imaging in the S1 File.

Another limitation of the APTw sequence is the relatively short total saturation transfer length of 744 ms (of which only 500 ms radio-frequency irradiation) resulting in the contrast to noise being lower compared to what can be expected with a longer saturation time. Blood vessels (high in protein) seem to be more highlighted for this setting. The gradient echo sequence is also not optimal in SNR compared to for instance the TSE sequence often used in the literature. An additional limitation is patient movement that might affect the quality. However, the most important general limitation for CEST/APT studies is that different pulse sequences for saturation will give different APTw signal intensity values, making it more difficult to compare absolute intensity effects for APT work from different groups and work performed on different scanners. For instance, the analysis done here was an integral over the 3.5 ± .4 ppm range compared to single frequency analysis in most papers in the literature. While most likely very similar, to achieve comparable contrast magnitudes between groups, there is a need for harmonization of scan parameters between investigators and ultimately manufacturers, as stipulated in recent reviews [16,25]. Finally, the analysis used in APTw MRI is a difference method that mixes multiple effects in the saturation spectrum of water [17,18,31], namely APT, nuclear Overhauser enhancements of mobile proteins (rNOEs) and asymmetry in the semi-solid magnetization transfer contrast (MTC). When using a different saturation scheme, the relative contributions of these effects change. For the current scheme, with B1 = 2μT but short saturation, we are in a range that the contribution from MTC asymmetry is less than in previous papers, which enhances the contrast from vascular proteins and the increase in tumors is likely to have a large blood-based component.

## Conclusion

We conclude that amide proton transfer weighted imaging (APTw imaging) in the clinical pre-surgical assessment efficiently helps the radiologist in differentiating LGGs from HGGs, which potentially improves patient management and treatment. Mean and max APTw signal intensities as individual metrics showed the greatest promise but also, a multibiometric approach (mean, max and range) trumped the sensitivity of any single biometric as 100% sensitivity and 87.5% specificity could be obtained in our glioma cohort.

## Supporting information

S1 Fig ROC curve for mean and max APTw signal and also the combined APTw mean and maximum signal by logistic regression. AUC, 95% CI, Sensitivity and specificity with cut off values are reported in S4 Table.
(DOCX)

S2 Fig APTw, T1-MPRAGE with Gadolinium axial, coronal, sagittal, FLAIR coronal and sagittal. Subject 3 with Low Grade Glioma, Astrocytoma WHO Grade 2 (LGG).
(DOCX)

S3 Fig APTw, T1-MPRAGE with Gadolinium axial, coronal, sagittal, FLAIR coronal and sagittal. Subject 1 with High Grade Glioma, Glioblastoma WHO Grade 4 (HGG).
(DOCX)

S4 Fig APTw, T1-MPRAGE with Gadolinium axial, coronal, sagittal, FLAIR coronal and sagittal. Subject 25 with metastasis, adenocarcinoma (MET).
(DOCX)

S1 Table Descriptive statistics of mean, max, min, range APTw-signal in % across HGG, LGG, MET. (DOCX)

S2 Table Mean and sum of ranks for HGG/LGG and APTw signals mean, max, min, range. (DOCX)

S3 Table Mann-Whitney U test for distinguishing low grade glioma and high grade glioma using APTw signal. (DOCX)

S4 Table ROC analysis for distinguishing HGG and LGG using APTw signal intensity. (DOCX)

S5 Table Classification of brain lesions into HGG/LGG by logistic regression models based on ROI encompassing whole tumor on 1 slice T1w+Gd MRI (APTw signals mean and maximum) vs. ROI with 10 pixels (APTw signals mean, maximum and range). (DOCX)

S1 File (ZIP)

## Abbreviations

LGG: Low Grade Glioma
HGG: High Grade Glioma
MET: Metastasis
APT: Amide Proton Transfer
APTw: Amide Proton Transfer weighted
WHO: World Health Organization
GBM: Glioblastoma Multiforme
GB: Glioblastoma
Gd: Gadolinium
MGMT: O6-methylguanine-DNA methyltransferase
IDH: Isocitrate Dehydrogenase
FLAIR: Fluid Attenuation Inversion Recovery
MPRAGE: Magnetization Prepared Rapid Acquisition Gradient Echo
NOE/rNOEs: Nuclear Overhauser Effect
MTC: Magnetization Transfer Contrast
STUPP: Treatment Protocol Named after Roger Stupp
GTR: Gross Total Resection
OS: Overall Survival
