# Correction: Assessment of Amide proton transfer weighted (APTw) MRI for pre-surgical prediction of final diagnosis in gliomas

**DOI:** 10.1371/journal.pone.0250189

**Published:** 2021-04-08

**Authors:** The PLOS ONE Staff

The captions for Figs 6, 7, and 8 are incorrectly switched. The caption that appears for Fig 6 should appear for Fig 8. The caption that appears for Fig 7 should appear for Fig 6. The caption that appears for Fig 8 should appear for Fig 7. The publisher apologizes for the error. Please see the correct captions for Figs 6, 7, and 8 here.

**Fig 6:**
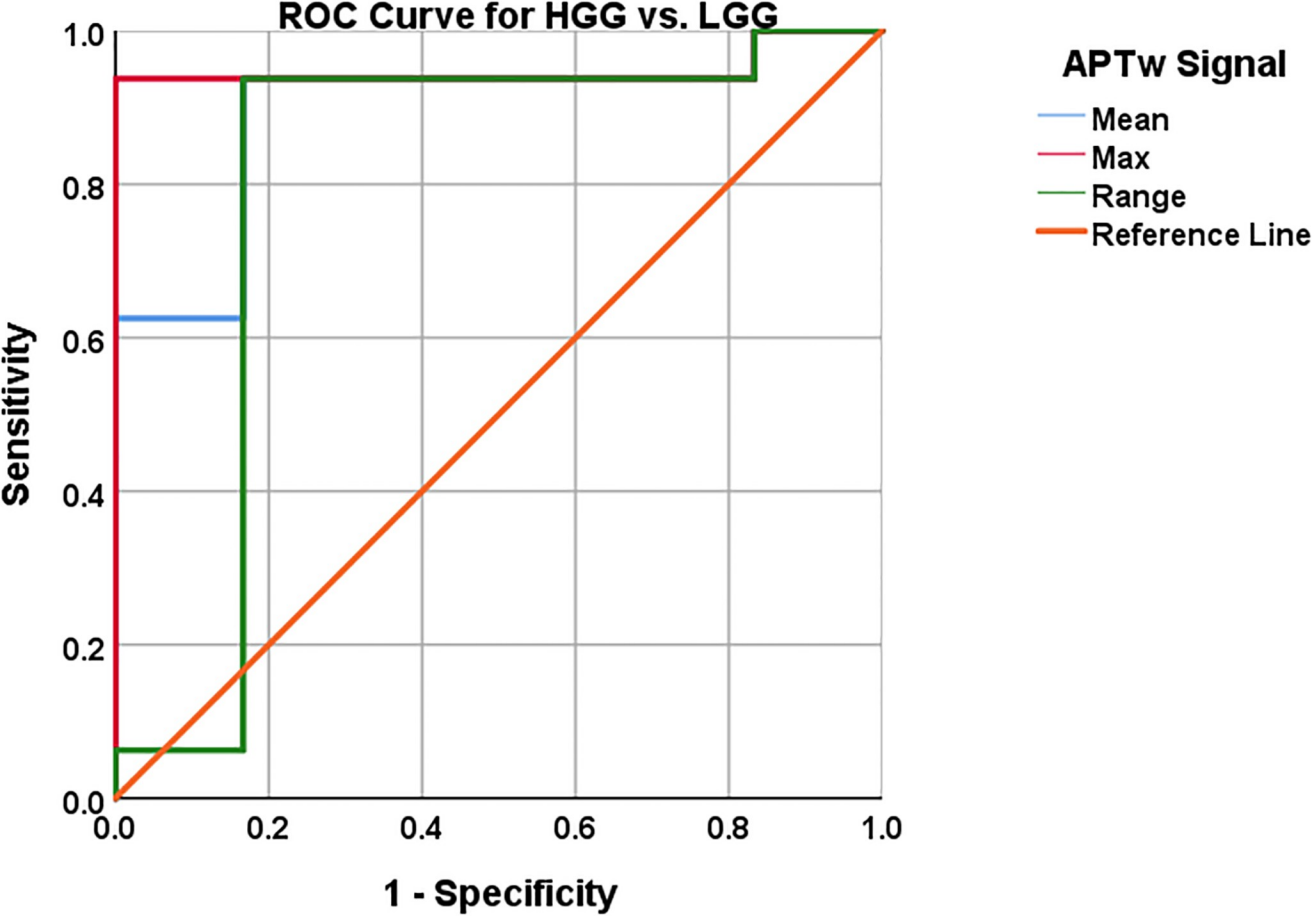
AUC, 95% CI, sensitivity and specificity with cut off values reported in Table 4.

**Fig 7:**
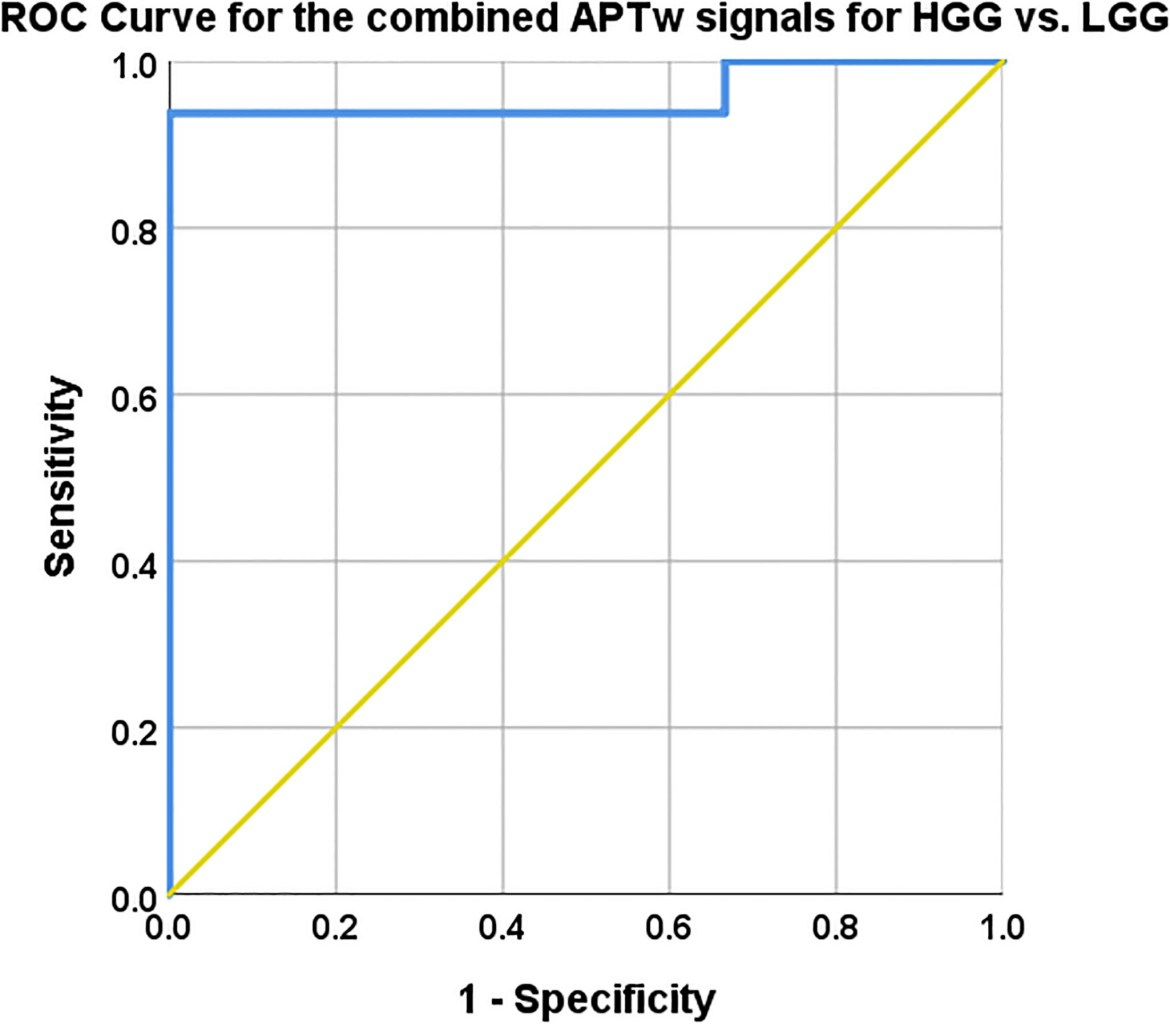
Mean, max and range APTw signal combined with logistic regression. AUC, 95% CI, Sensitivity and specificity with cut off values reported in Table 4. The combined model mislabelled subjects 3 and 17 as they were labelled HGG in the model but are histologically verified LGG, also subject 7 was mislabelled as a LGG whereas it is histologically a Glioblastoma, Table 1.

**Fig 8:**
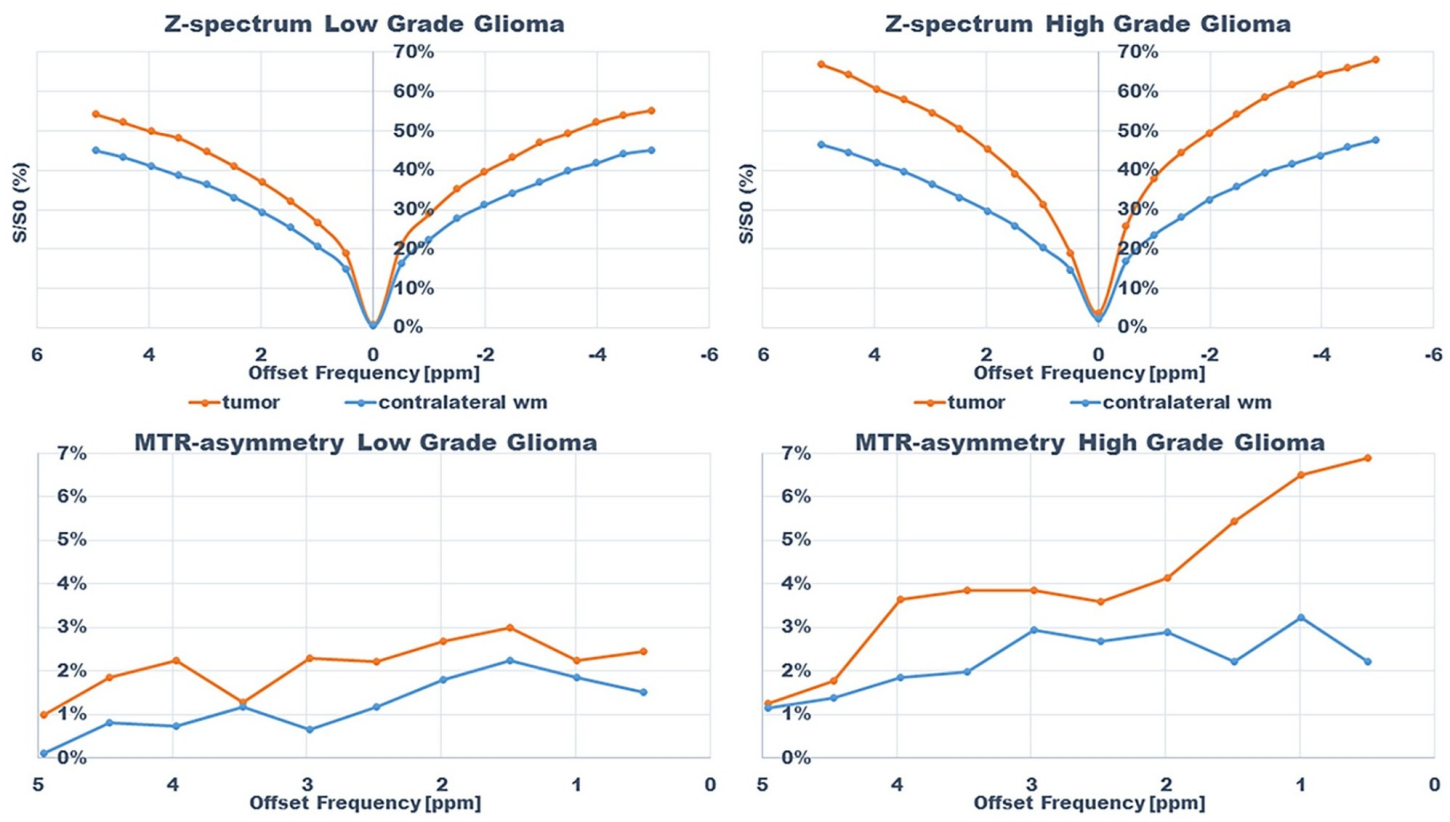
Z-spectra and magnetization transfer ratio asymmetry spectra for subjects; subject 13 (HGG) and 9 (LGG) within tumor and in contralateral normal appearing white matter.
