# Supplementary material for: Assessment of Amide proton transfer weighted (APTw) MRI for pre-surgical prediction of final diagnosis in gliomas
Source: PLoS One. 2020 Dec 29;15(12):e0244003. doi: 10.1371/journal.pone.0244003 (PMC7771875; doi:10.1371/journal.pone.0244003)
Supplement: S1 Fig — AUC, 95% CI, Sensitivity and specificity with cut off values are reported in S4 Table. (DOCX) [file pone.0244003.s001.docx]

Fig S1.1


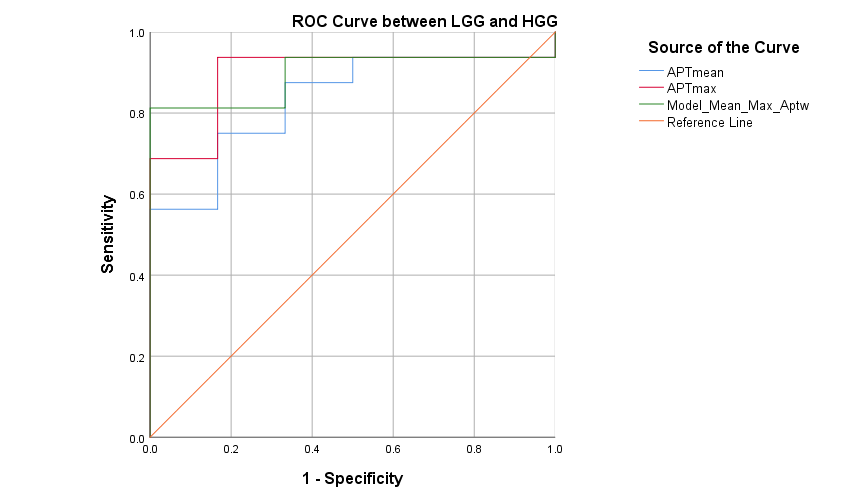
Legend for Fig S1.1
ROC curve for mean and max APTw signal and also the combined APTw mean and maximum signal by logistic regression. AUC, 95 % CI, Sensitivity and specificity with cut off values are reported in Table S1.4.
