# Supplementary material for: Assessment of Amide proton transfer weighted (APTw) MRI for pre-surgical prediction of final diagnosis in gliomas
Source: PLoS One. 2020 Dec 29;15(12):e0244003. doi: 10.1371/journal.pone.0244003 (PMC7771875; doi:10.1371/journal.pone.0244003)
Supplement: S2 Fig — Subject 3 with Low Grade Glioma, Astrocytoma WHO Grade 2 (LGG). (DOCX) [file pone.0244003.s002.docx]

Fig S1.2


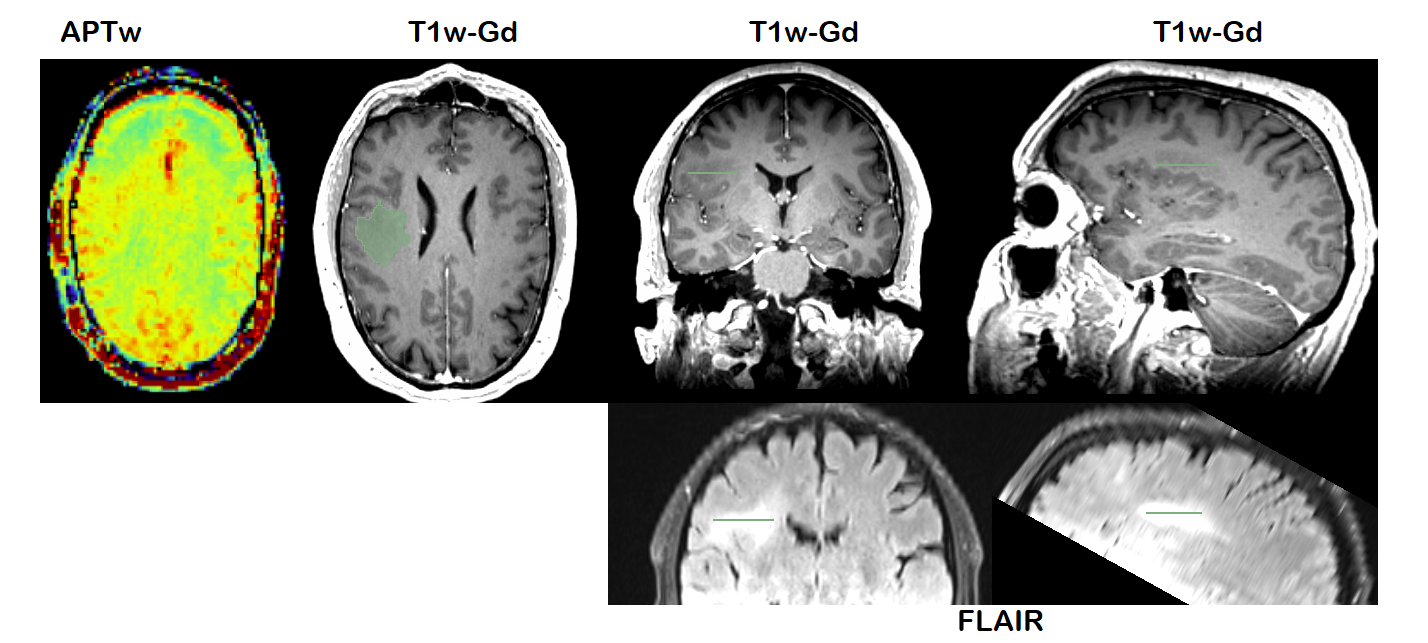


Legend for Fig S1.2
APTw, T1-MPRAGE with Gadolinium axial, coronal, sagittal, FLAIR coronal and sagittal. Subject 3 with Low Grade Glioma, Astrocytoma WHO Grade 2 (LGG).
