# Supplementary material for: Assessment of Amide proton transfer weighted (APTw) MRI for pre-surgical prediction of final diagnosis in gliomas
Source: PLoS One. 2020 Dec 29;15(12):e0244003. doi: 10.1371/journal.pone.0244003 (PMC7771875; doi:10.1371/journal.pone.0244003)
Supplement: S1 Table — (DOCX) [file pone.0244003.s005.docx]

Supplementary Material Tables

Table S1.1
Descriptive statistics of mean, max, min, range APTw-signal in % across HGG, LGG, MET

| **HGG (n=16)** | Mean APTw % | Max APTw % | Min APTw % | Range APTw % |
| --- | --- | --- | --- | --- |
| Average | 2.28 | 3.18 | 1.36 | 1.82 |
| Minimum | 0.95 | 1.65 | 0.14 |  |
| Maximum | 3.98 | 5.26 | 3.34 |  |
| Range | 3.03 | 3.61 | 3.20 |  |
| **LGG (n=6)** |  |  |  |  |
| Average | 1.60 | 2.22 | 0.90 | 1.32 |
| Minimum | 1.20 | 1.94 | 0.32 |  |
| Maximum | 2.13 | 2.60 | 1.74 |  |
| Range | 0.93 | 0.66 | 1.42 |  |
| **MET (n=2)** |  |  |  |  |
| Average | 2.17 | 2.82 | 1.45 | 1.37 |
| Minimum | 1.94 | 2.77 | 0.90 |  |
| Maximum | 2.39 | 2.87 | 2.00 |  |
| Range | 0.45 | 0.10 | 1.10 |  |
