# Supplementary material for: Assessment of Amide proton transfer weighted (APTw) MRI for pre-surgical prediction of final diagnosis in gliomas
Source: PLoS One. 2020 Dec 29;15(12):e0244003. doi: 10.1371/journal.pone.0244003 (PMC7771875; doi:10.1371/journal.pone.0244003)
Supplement: S2 Table — (DOCX) [file pone.0244003.s006.docx]

Table S1.2 Mean and Sum of Ranks for HGG/LGG and APTw Signals Mean, Max, Min, Range.

| **Ranks** | | | | |
| --- | --- | --- | --- | --- |
|  | Group | N | Mean Rank | Sum of Ranks |
| APTmean | HGG | 16 | 13.50 | 216.00 |
|  | LGG | 6 | 6.17 | 37.00 |
|  | Total | 22 |  |  |
| APTmax | HGG | 16 | 13.88 | 222.00 |
|  | LGG | 6 | 5.17 | 31.00 |
|  | Total | 22 |  |  |
| APTmin | HGG | 16 | 12.38 | 198.00 |
|  | LGG | 6 | 9.17 | 55.00 |
|  | Total | 22 |  |  |
| APTrange | HGG | 16 | 12.53 | 200.50 |
|  | LGG | 6 | 8.75 | 52.50 |
|  | Total | 22 |  |  |
