# Supplementary material for: Assessment of Amide proton transfer weighted (APTw) MRI for pre-surgical prediction of final diagnosis in gliomas
Source: PLoS One. 2020 Dec 29;15(12):e0244003. doi: 10.1371/journal.pone.0244003 (PMC7771875; doi:10.1371/journal.pone.0244003)
Supplement: S3 Table — (DOCX) [file pone.0244003.s007.docx]

Table S1.3 Mann-Whitney U test for distinguishing Low Grade Glioma and High Grade Glioma using APTw signal

| **Mann Whitney U test Between LGG and HGG** | | | | |
| --- | --- | --- | --- | --- |
|  | APTmean | APTmax | APTmin | APTrange |
| Mann-Whitney U | 16.0 | 10.0 | 34.0 | 31.5 |
| P-value = | .018 | .005 | .302 | .224 |
| *Note that IDH- and MGMT promoter methylation status was not significantly different across APTw signal for LGG and HGG nor between HGG when tested within group. | | | | |
