# Supplementary material for: Assessment of Amide proton transfer weighted (APTw) MRI for pre-surgical prediction of final diagnosis in gliomas
Source: PLoS One. 2020 Dec 29;15(12):e0244003. doi: 10.1371/journal.pone.0244003 (PMC7771875; doi:10.1371/journal.pone.0244003)
Supplement: S4 Table — (DOCX) [file pone.0244003.s008.docx]

Table S1.4 ROC analysis for distinguishing HGG and LGG using APTw signal intensity

|  |  | | | | | | |
| --- | --- | --- | --- | --- | --- | --- | --- |
| APTw signal | | Area Under the Curve AUC | P-value = | 95% Confidence Interval | |  |  |
|  |  |  |  | Lower Bound | Upper Bound | Cutoff / Sensitivity / Specifcity |  |
| Mean | | .833 | .018 | .658 | 1.000 | 1.73% / 87.5% / 67.7% |  |
| Max | | .896 | .005 | .752 | 1.000 | 2.38% / 93.8% / 83.3% |  |
| Combined* | | .896 | .005 | .758 | 1.000 | 0.34 / 81.3% / 100% |  |

*Logistic Regression Model with combined Mean and Max APTw signal
