# Supplementary material for: Assessment of Amide proton transfer weighted (APTw) MRI for pre-surgical prediction of final diagnosis in gliomas
Source: PLoS One. 2020 Dec 29;15(12):e0244003. doi: 10.1371/journal.pone.0244003 (PMC7771875; doi:10.1371/journal.pone.0244003)
Supplement: S5 Table — (DOCX) [file pone.0244003.s009.docx]

Table S1.5 Classification of brain lesions into HGG/LGG by logistic regression models based on ROI encompassing whole tumor on 1 slice T1w+Gd MRI (APTw signals mean and maximum) vs. ROI with 10 pixels (APTw signals mean, maximum and range)

| Subject | Predicted Group Membership -Whole tumor slice | Predicted Group Membership -10 pixel ROI |
| --- | --- | --- |
| 1 | Correct | Correct |
| 2 | Correct | Correct |
| 3 | Correct | Incorrect (LGG) |
| 4 | Correct | Correct |
| 5 | Correct | Correct |
| 6 | Correct | Correct |
| 7 | Incorrect (HGG) | Incorrect (HGG) |
| 8 | Correct | Correct |
| 9 | Correct | Correct |
| 10 | Correct | Correct |
| 11 | Incorrect (LGG) | Correct |
| 13 | Correct | Correct |
| 14 | Correct | Correct |
| 15 | Correct | Correct |
| 17 | Correct | Incorrect (LGG) |
| 18 | Correct | Correct |
| 19 | Correct | Correct |
| 20 | Correct | Correct |
| 21 | Correct | Correct |
| 22 | Correct | Correct |
| 23 | Correct | Correct |
| 24 | Incorrect (LGG) | Correct |

*Incorrectly labeled lesion, within parenthesis is the correct diagnosis.
